# Practical, sustainable, wide-temperature-adaptable zinc-metal batteries enabled by electrogelated recyclable biomacromolecular hydrogel electrolytes

**DOI:** 10.1093/nsr/nwaf308

**Published:** 2025-07-31

**Authors:** Jing Huang, Sijun Wang, Le Yu, Erlantz Lizundia, Xuanyu Zeng, Lu Chen, Ziyang Lu, Luhe Qi, Yu Lin, Hongbing Deng, Chaoji Chen

**Affiliations:** Hubei Biomass-Resource Chemistry and Environmental Biotechnology Key Laboratory, Hubei Provincial Engineering Research Center of Emerging Functional Coating Materials, School of Resource and Environmental Sciences, Wuhan University, Wuhan 430079, China; Hubei Biomass-Resource Chemistry and Environmental Biotechnology Key Laboratory, Hubei Provincial Engineering Research Center of Emerging Functional Coating Materials, School of Resource and Environmental Sciences, Wuhan University, Wuhan 430079, China; Hubei Biomass-Resource Chemistry and Environmental Biotechnology Key Laboratory, Hubei Provincial Engineering Research Center of Emerging Functional Coating Materials, School of Resource and Environmental Sciences, Wuhan University, Wuhan 430079, China; Life Cycle Thinking Group, Department of Graphic Design and Engineering Projects, University of the Basque Country (UPV/EHU), Bilbao 48013, Spain; Hubei Biomass-Resource Chemistry and Environmental Biotechnology Key Laboratory, Hubei Provincial Engineering Research Center of Emerging Functional Coating Materials, School of Resource and Environmental Sciences, Wuhan University, Wuhan 430079, China; Hubei Biomass-Resource Chemistry and Environmental Biotechnology Key Laboratory, Hubei Provincial Engineering Research Center of Emerging Functional Coating Materials, School of Resource and Environmental Sciences, Wuhan University, Wuhan 430079, China; Hubei Biomass-Resource Chemistry and Environmental Biotechnology Key Laboratory, Hubei Provincial Engineering Research Center of Emerging Functional Coating Materials, School of Resource and Environmental Sciences, Wuhan University, Wuhan 430079, China; Hubei Biomass-Resource Chemistry and Environmental Biotechnology Key Laboratory, Hubei Provincial Engineering Research Center of Emerging Functional Coating Materials, School of Resource and Environmental Sciences, Wuhan University, Wuhan 430079, China; Hubei Biomass-Resource Chemistry and Environmental Biotechnology Key Laboratory, Hubei Provincial Engineering Research Center of Emerging Functional Coating Materials, School of Resource and Environmental Sciences, Wuhan University, Wuhan 430079, China; Hubei Biomass-Resource Chemistry and Environmental Biotechnology Key Laboratory, Hubei Provincial Engineering Research Center of Emerging Functional Coating Materials, School of Resource and Environmental Sciences, Wuhan University, Wuhan 430079, China; Hubei Biomass-Resource Chemistry and Environmental Biotechnology Key Laboratory, Hubei Provincial Engineering Research Center of Emerging Functional Coating Materials, School of Resource and Environmental Sciences, Wuhan University, Wuhan 430079, China

**Keywords:** biomacromolecule, aqueous zinc-metal batteries, hydrogel electrolytes, wide-temperature, electrofabrication

## Abstract

In the promotion of aqueous zinc (Zn)-metal batteries, hydrogel electrolytes have been found to come with the capability to physically obstruct dendritic Zn growth and suppress unwanted side reactions compared to their liquid counterparts. However, due to the opposite structural requirements for ionic conductivity and mechanical strength, the preparation of thin-film hydrogel electrolytes for energy-dense Zn-metal batteries remains a formidable task. Herein, electrogelation that offers several unique advantages, including precise control over the thickness, good geometric adaptability, and high tunability of the hydrogels’ microstructures and properties, is investigated as a promising technique to address the above-mentioned dilemma. It is shown that, by varying the electrogelation parameters, various biomacromolecular hydrogel electrolytes featuring highly micro-/nanostructured pores can be obtained to deliver simultaneously high mechanical strength (2.0 to 4.4 MPa) and high ionic conductivity (10.1 to 19.5 mS cm^−1^). Such *in situ*–built electrolytes can be as thin as 50 μm and enable excellent Zn plating/stripping reversibility at 1 mA cm^−2^/1 mAh cm^−2^ with Coulombic efficiency averaging 99.83% for over 2800 cycles (>6 months); the Zn-metal battery with a high areal capacity of 5.4 mAh cm^−2^ and a practical negative-to-positive capacity ratio of 1.1 retains 70% of its initial capacity after 120 cycles. We quantitatively show the respectable recyclability, low environmental impact, and cost-effectiveness of our biomacromolecular hydrogel electrolytes by integrating experimental study, life-cycle assessment, and techno-economic analysis, thereby opening the door for research on more sustainable Zn-metal batteries and beyond.

## Teaser

In situ electrogelated biomacromolecular hydrogel electrolytes enable zinc metal batteries to operate reliably with prolonged cycle life across a wide temperature range. Being greenly fabricated and easily recyclable, they provide a sustainable and economically viable route towards closed-loop energy storage technologies.

## INTRODUCTION

Among various alternative energy storage technologies for large-scale energy storage applications, aqueous zinc (Zn)-metal batteries (AZMBs) have sparked tremendous research activity due to their intrinsic safety and cost-effectiveness of both metallic Zn and aqueous electrolyte [1,2], and concurrently reasonable energy density afforded by a Zn anode that features a high theoretical specific capacity of 820 mAh g^−1^ (or 5855 mAh cm^−3^). Despite significant progress in recent years, the commercialization of the AZMBs has still been plagued by the Zn dendrite issue and parasitic reactions (including Zn corrosion and hydrogen evolution reaction (HER)) associated with conventional aqueous electrolytes [3].

Compared with liquid electrolytes, polymer hydrogel electrolytes typically have lower water reactivity due to the extensive formation of water-polymer bonding interactions, making them less susceptible to HER and Zn corrosion [4,5]. Additionally, the polymer networks of hydrogel electrolytes are uniform at the molecular scale, which allows for evenly distributed Zn^2+^ flux at the Zn anode surface, thereby facilitating smoother Zn deposition compared to conventional liquid electrolyte/separator systems [6]. These advantages render hydrogel electrolytes a more promising candidate for AZMBs. However, to date, preparing thin-film hydrogel electrolytes that possess both superior mechanical performance and high ionic conductivity remains rather challenging. Most of the reported hydrogel electrolytes are characterized by low polymer content in order to retain reasonably high ionic conductivities. Such hydrogels usually suffer from weak mechanical strength. This makes it difficult to process them into thin films that are suitable for practical AZMB applications. Additionally, traditional hydrogel electrolytes are predominantly fabricated by crosslinking petroleum-derived monomers in aqueous media, making recycling challenging from an environmental perspective. Fortunately, the utilization of naturally occurring and biodegradable biomass materials constitutes a sustainable option as it can considerably offset these negative environmental consequences [5]. Hence, innovations in the fabrication of biomacromolecular hydrogel electrolytes with suitably engineered microstructures to optimize the trade-off between mechanical properties and ionic conductivity may be the final solution for practical, low-cost, and environmentally sustainable AZMBs.

**Figure 1.**
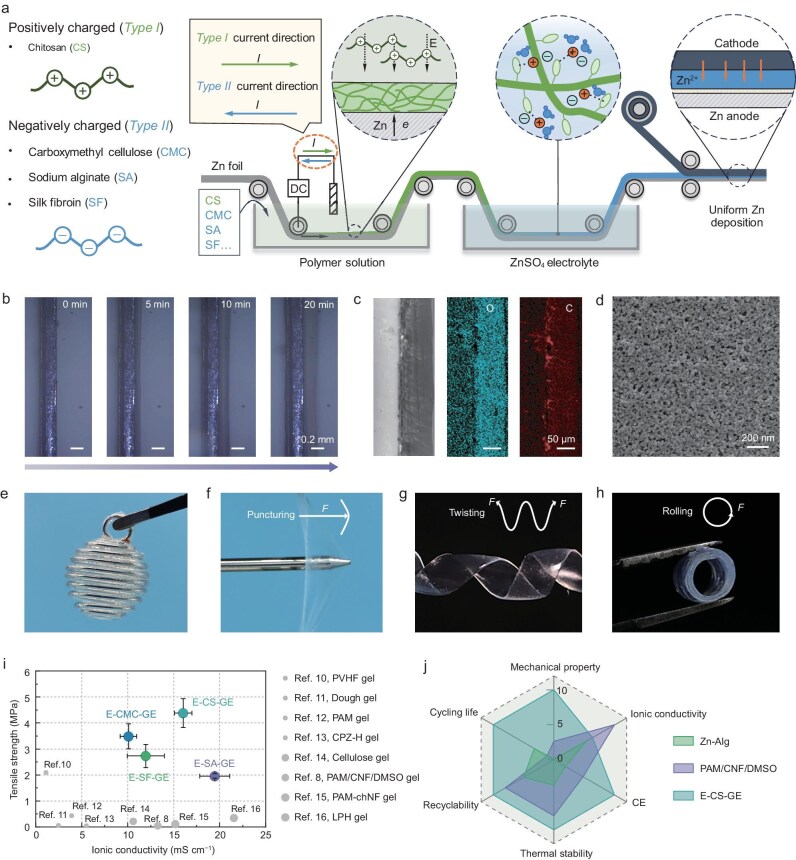
Schematic illustration and characteristics of strong biomacromolecular hydrogel electrolyte via *in situ* electrogelation. (a) Schematic diagram of the *in situ* electrogelation approach to synthesize strong hydrogel electrolytes for uniform Zn deposition. (b) Optical microscope images showing the formation of E-CS-Gel on Zn foil during the electrogelation process. (c) SEM images with elemental distributions. (d) Magnified image of the E-CS-Gel on Zn foil. (e) Optical photographs of E-CS-GE complex-shaped substrate. (f–h) Optical photographs of punctured (f), twisted (g), and rolled E-CS-GE (h). (i) Comparison of tensile strength and ionic conductivity of the E-GE with the previously reported gel electrolytes. (j) Radar plot of properties and performance of reported hydrogel electrolytes of Zn-Alg (Zn-alginate polymer electrolyte) [7], PAM/CNF/DMSO (polyacrylamide/cellulose nanofiber/dimethyl sulfoxide electrolytes) [8], and the E-CS-GE developed in this work. The ratings between 1 and 10 are normalized values based on the measured properties/performance of each electrolyte.

In this study, we demonstrate the electrogelation technique as a powerful tool for the *in situ* fabrication of sustainable, recyclable, and wide-temperature-adaptable biomacromolecular hydrogel electrolytes with a combination of high mechanical strength, ionic conductivity, and low carbon footprint (Fig. 1). As described in Fig. 1a, in specific aqueous solutions where macromolecules are positively or negatively charged, an appropriate external electric field can induce directional transport and occurrence of *in situ* sol-gel transition of these macromolecules onto the Zn anode side. After undergoing an aqueous electrolyte immersion process, the resultant electrogelated hydrogel electrolyte (E-GE)/Zn anode integration can be directly applied to the battery assembly. The whole preparation process is highly feasible and practical, specifically reflected in the following two aspects: (i) the thickness and microstructure of the electrolyte are tunable by varying the reaction time and applied electric field; (ii) the high conformality of hydrogel electrolytes prepared by the electrogelation method enables these electrolytes to be applied to electrode substrates with complex geometries. Investigations on the best-performing E-GE prepared from chitosan (denoted as E-CS-GE) suggest that the unique combination of high mechanical strength and sufficient ionic conductivity is afforded by the uniform nanoporous CS network structure. Through combined experimental and computational studies, we demonstrate that E-CS-GE manifests significant advantages in the combination of mechanical property and ionic conductivity, Zn^2+^ plating/stripping reversibility, cycling life, thermal stability, and recyclability (Fig. 1j and Table S1) [7,8]. The generality of the electrogelation method was further demonstrated by the enhanced Zn cycling longevity enabled by E-GE prepared from other biomacromolecules, including carboxymethyl cellulose (CMC), sodium alginate (SA), and silk fibroin (SF).

## RESULTS AND DISCUSSION

### *In situ* electrogelation and characterizations of E-GE

Typically, the *in situ* electrogelation process involves electrophoretic transport of the charged biopolymers (the positively charged CS or negatively charged ones like CMC, SA, and SF) towards the Zn electrode and hydrogel formation on the electrode surface (Fig. 1a). More specifically, taking the case of CS as an example, as shown in Fig. S1, the pH-dependent solubility renders CS with interesting physicochemical properties: the primary amino groups of CS have an isoelectric point (pI) value of ∼6.3 [9]. Below this value, the amino groups are protonated, making CS a soluble cationic polyelectrolyte; conversely, it becomes insoluble because the amino groups are deprotonated. When an external current is imposed, the local pH in the vicinity of the Zn electrode increases as the release of OH^−^ through the reaction H_2_O_2_ + 2e^−^ → 2OH^−^, accompanied by the electrogelation of CS on the Zn surface. The reaction kinetics is governed by electrodiffusion of OH^−^ and protonated CS-NH_3_^+^ polymers with a positive zeta potential of 50.6 mV (Fig. S2). As recorded by the optical images (Fig. 1b and Fig. S3), the thickness of the electrogelated CS hydrogel (E-CS-Gel) increases almost linearly with operating time. A 200-µm-thick hydrogel film was successfully built onto a Zn surface within 20 min. A large-sized (100 × 15 cm^2^) E-CS-GE was prepared using a custom-made semi-continuous electrogelation device, confirming the scalability of our electrogelation method (Fig. S4). Morphological and compositional examination of the E-CS-Gel by field emission scanning electron microscopy (FE-SEM) and energy-dispersive X-ray spectroscopy (EDS) confirms the successful preparation of E-CS-Gel with uniformly-distributed nanopores with an average size of ∼10 nm (Fig. 1c and d, and Fig. S5). Moreover, the excellent conformability of the electrogelation method allows for superior interfacial contact and strong adhesion, as well as the introduction of an E-CS-GE layer with three-dimensional (3D) complex geometries (Fig. 1e and Fig. S6), which could hardly be realized through other conventional hydrogel preparation methods. E-GEs based on negatively charged macromolecules could be prepared using a similar process, a notable difference is that the direction of applied current is the opposite (see Fig. S7 for more details).

Among various mechanical parameters of gel polymer electrolytes (GPEs), tensile strength and toughness are of the greatest significance for addressing dendrite issues and facilitating battery assembly and operation. Deformation tests by hand suggest all four E-GEs—E-CMC-GE (prepared from CMC), E-SA-GE (prepared from SA), E-SF-GE (prepared from SF), and E-CS-GE—have good flexibility with a certain extent of stretchability, and can repeatedly withstand severe deformations of twisting, rolling, and especially puncturing (Fig. 1f–h and Fig. S8). Further, uniaxial tensile stress-strain tests were performed to quantitatively determine the tensile properties of the four E-GEs. As shown in Fig. S9, the tensile strengths of E-CMC-GE, E-SA-GE, E-SF-GE, and E-CS-GE are ∼3.5, 2.0, 2.7, and 4.4 MPa, respectively, with maximum strains of 33.9%, 135.8%, 25.9%, and 102.2%. The corresponding toughness values are 0.86, 1.93, 0.58, and 1.94 MJ m^−3^. The superior mechanical properties are attributed to the uniform and dense nanoporous structure and the lower water content of the E-GEs (Fig. 1d, Figs S9 and S10). Such mechanical performance basically meets the requirements for practical batteries. Historically, achieving high mechanical strength for GPEs was done at the expense of ionic conductivity. In this work, unexpectedly, the ionic conductivities reach 10.1 mS cm^−1^ for E-CMC-GE, 19.5 mS cm^−1^ for E-SA-GE, 12.0 mS cm^−1^ for E-SF-GE, and 16.0 mS cm^−1^ for E-CS-GE, as determined by electrochemical impedance spectroscopy (EIS) (Fig. S11). The decoupling of mechanical performance and ionic conductivity is partially attributed to highly ordered microporous and nanoporous matrix structures formed through a constant electrogelation process, as shown in Fig. 1d and Fig. S7. Additionally, the interactions between these selected biomacromolecules and the ionic species may also be responsible for the enhanced ion transport kinetics, which will be discussed later. Figure 1i summarizes the tensile strength versus ionic conductivity of hydrogel electrolytes for Zn batteries developed both in this work and literature (original data shown in Table S2) [8,10–16]. The four electrolytes developed in this work exhibited a unique combination of high tensile strength (2.0–4.4 MPa) and sufficient ionic conductivity (10.1 to 19.5 mS cm^−1^), outperforming all reported hydrogel electrolytes for Zn batteries to date. Among the four E-GEs, E-CS-GE was selected for further research due to its uniqueness of having the highest toughness of 1.94 MJ m^−3^ and the second highest ionic conductivity of 16.0 mS cm^−1^, which is most compelling for battery applications.

### Evaluation of Zn plating/stripping performance enabled by the E-CS-GE

**Figure 2.**
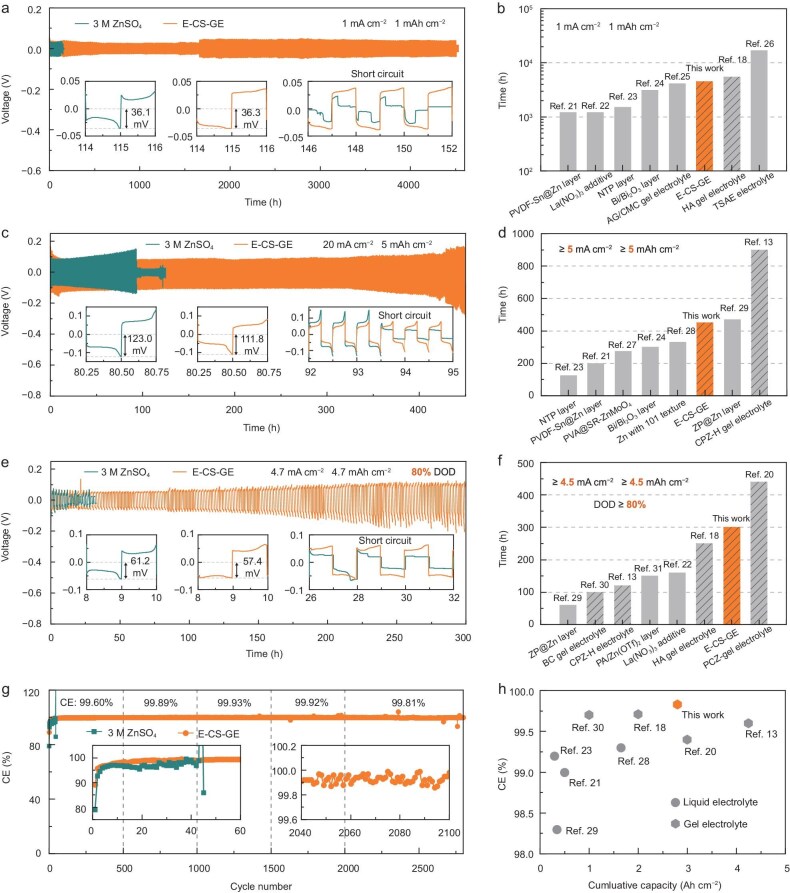
Electrochemical performance of symmetric and asymmetric cells using 3 M ZnSO_4_ and *in situ* E-CS-GE. (a–f) The cycling performance of Zn||Zn symmetric cells. Galvanostatic Zn plating/stripping in Zn||Zn symmetric cells using 3 M ZnSO_4_ and E-CS-GE at 1 mA cm^−2^ with a capacity of 1 mAh cm^−2^ (a), 20 mA cm^−2^ and 5 mAh cm^−2^ (c), and high DOD of 80% at 4.7 mA cm^−2^ and 4.7 mAh cm^−2^ (e). Comparison of the cycling life at the current density and capacity of 1 mA cm^−2^ and 1 mAh cm^−2^ (b), ≥5 mA cm^−2^ and ≥5 mAh cm^−2^ (d), and with a DOD ≥80% between this work and previous studies (f). The insets are the enlarged view of Zn plating/stripping curves. (g) CEs of Zn||Cu asymmetric cells with 3 M ZnSO_4_ and E-CS-GE versus cycle number, tested at 1 mA cm^−2^ and 1 mAh cm^−2^ (upper cut-off voltage: 0.8 V). (h) Average CE versus cumulative capacity for Zn||Cu cells in this work and previous studies. The solid and hatched bars in (b, d and f) represent liquid and gel electrolytes, respectively.

Long-term galvanostatic cycling tests of Zn||Zn symmetric cells were first performed using the widely accepted test protocol of 1 mA cm^−2^/1 mAh cm^−2^. As shown in Fig. 2a, the E-CS-GE (200 μm in thickness in the undeformed state if not otherwise specified) enables an ultralong cycle life of >4500 h (∼6 months), which is ∼30 times longer than that of the cell with 3 M ZnSO_4_ as the control electrolyte. The overpotentials are low and stable, outperforming those reported in hydrogel electrolytes for AZMBs (Table S3), [12,14,17–20] suggesting a fast Zn plating/stripping kinetics and Zn/electrolyte interface stability afforded by the E-CS-GE. Even when the electrolyte thickness is reduced to challenging levels of 50 and 100 μm from a practical application perspective, Zn||Zn symmetric cells with the E-CS-GE withstand over 1400 and 2300 h of cycling, respectively (Fig. S12). This stability surpasses most existing hydrogel electrolytes with comparable thicknesses. To the best of our knowledge, the Zn cycling life of >4500 h enabled by our E-CS-GE ranks third among all reported hydrogel electrolytes to date (Fig. 2b and Table S4) [18,21–26].

At a significantly higher current density of 20 mA cm^−2^ and a practical-level areal capacity of 5 mAh cm^−2^, the overpotential of a Zn symmetric cell with 3 M ZnSO_4_ electrolyte experiences a continuous increase to 143.7 mV before short-circuit, showing poor Zn/electrolyte interface stability and therefore a short lifespan of only 94 h, while the Zn symmetric cell with E-CS-GE can be operated for >450 h and delivers very stable overpotentials of ∼112 mV within the initial 400 h (Fig. 2c). Among the previously reported cycling performances of Zn symmetric cells using hydrogel electrolytes at a current density ≥5 mA cm^−2^ and an areal capacity ≥5 mAh cm^−2^ (Fig. 2d and Table S4) [13,21,23,24,27–29], the E-CS-GE is second only to the hydrogel electrolyte developed by Xu and co-workers. Using a more rigorous test protocol of ultra-thin Zn foils (10 μm, corresponding to a theoretical capacity of 5.85 mAh cm^−2^) and a high depth of discharge (DOD) of 80% (areal capacity of 4.7 mAh cm^−2^) to simulate the low negative/positive capacity (N/P) ratio conditions of practical AZMBs, in sharp contrast to the fast failure of the 3 M ZnSO_4_ cell within only 29 h, a remarkably long cycling lifetime of >300 h is achieved by the E-CS-GE (Fig. 2e and f and Table S4) [13,18,20,22,29–31]. Furthermore, Zn symmetric cells were subjected to sequential current density steps of 1 to 20 mA cm^−2^ to emulate dynamic rate scenarios in practical AZMBs. As shown in Fig. S13, the E-CS-GE–based cell exhibits stable Zn plating/stripping overpotentials of 36.7, 45.6, 79.8, 119.1 and 160.9 mV at 1, 2, 5, 10 and 20 mA cm⁻², respectively. These overpotentials are comparable to or marginally higher than those observed in the 3 M ZnSO₄ cell. Considering the ionic conductivity of the E-CS-GE is only approximately one-third that of 3 M ZnSO_4_ (16.0 vs 45.9 mS cm^−1^, Fig. S14), its significantly improved Zn^2+^ transference number of 0.28 ± 0.11 compared to 0.06 ± 0.01 for 3 M ZnSO_4_ (experimentally determined via the Bruce–Vincent method, Figs S15 and S16) is believed to primarily account for this exceptional rate capability.

Complementary to Zn symmetric cells, the ratio of stripped Zn to plated Zn on the Cu substrate in Zn||Cu asymmetric cells can quantitatively reflect the reversibility of the Zn anode. As shown in Fig. 2g, at a current density of 1 mA cm^−2^ and a constant plating areal capacity of 1 mAh cm^−2^, the Zn||Cu cell with E-CS-GE shows a favorable first-cycle Coulombic efficiency (CE) of 89.14% and can be stably operated with remarkably high CEs of ∼99.9% over subsets of cycles (99.89% of 500th to 1000th cycle, 99.93% of 1000th to 1500th cycle, and 99.92% of 1500th to 2000th cycle). As a result, an average CE of 99.83% (including the CE values of the initial formation cycles) is observed throughout the ultra-long cycle life of 2800 cycles. To the best of our knowledge, this represents the highest levels of Zn reversibility in terms of cumulative capacity and average CE ever achieved (Fig. 2h and Table S5) [13,18,20,21,23,28–30]. These results strongly indicate the excellent reversibility and fast kinetics of Zn plating/stripping enabled by the E-CS-GE. By contrast, Zn||Cu with the baseline electrolyte of 3 M ZnSO_4_ only survives for 43 cycles associated with a lower average CE of <97%. Considering the excellent Zn symmetric performances under practical conditions of wide current density range (1–20 mA cm^−2^), high areal capacity up to 5 mAh cm^−2^, high DOD of 80%, and a 99.83% CE for long-term Zn||Cu testing, our E-CS-GE stands out as one of the most promising electrolyte candidates for practical AZMBs.

### Mechanistic insights into the enhanced Zn cyclability enabled by E-CS-GE

**Figure 3.**
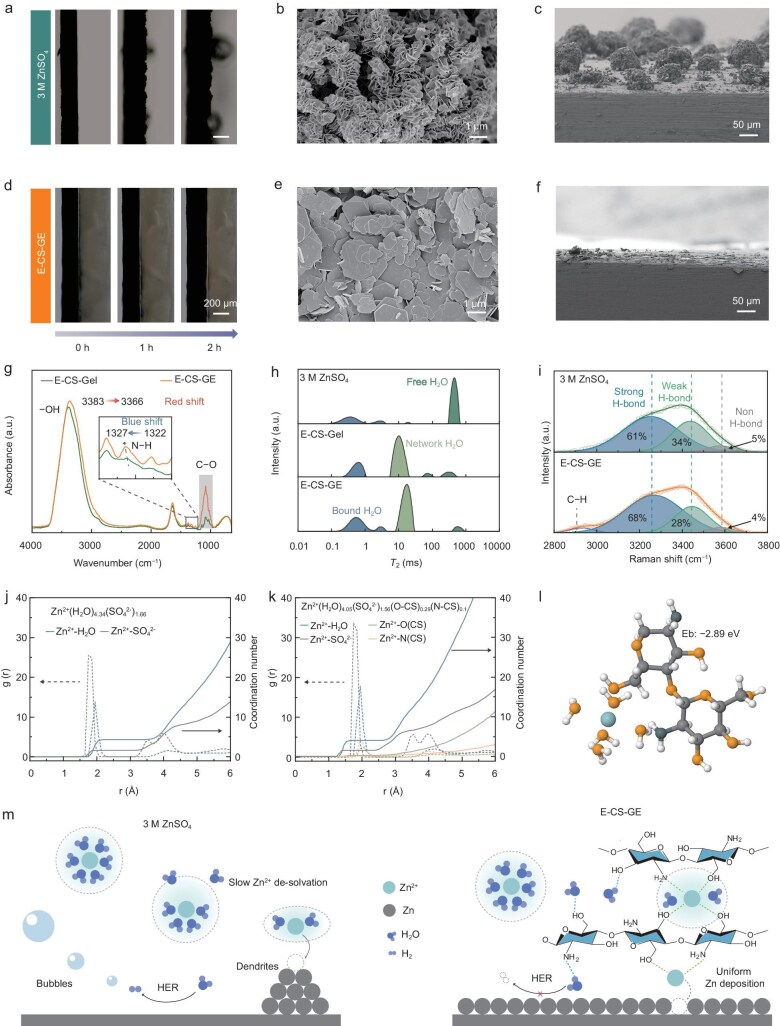
Mechanism investigation of the enhanced Zn cyclability enabled E-CS-GE. (a–f) Morphology of the Zn^2+^ electrodeposition process. *In situ* optical microscopy observation of Zn^2+^ electrodeposition in 3 M ZnSO_4_ (a) and E-CS-GE (d) under a current density of 1 mA cm^−2^. SEM images of Zn morphology after 20 cycles at 1 mA cm^−2^ and 1 mAh cm^−2^ in 3 M ZnSO_4_ from a top view (b) and cross-section view (c), and E-CS-GE from a top view (e) and cross-section view (f). (g) FTIR spectra of E-CS-Gel and E-CS-GE. (h) Distribution curves of transverse relaxation time spectra of 3 M ZnSO_4_, E-CS-Gel, and E-CS-GE. (i) Fitting results of the O−H stretching band in the Raman spectra for comparing the H-bonding states of water in 3 M ZnSO_4_ and E-CS-GE. (j and k) Radial distribution function *g*(*r*) and coordination number of Zn^2+^–H_2_O and Zn^2+^–SO_4_^2−^ in 3 M ZnSO_4_ (j), and Zn^2+^–H_2_O, Zn^2+^–SO_4_^2−^, Zn^2+^–O (CS), and Zn^2+^–N (CS) in E-CS-GE (k) obtained from MD simulation. (l) DFT computed binding energy to form the cluster of Zn^2+^·5H_2_O·C_14_O_8_H_24_. (m) Schematic illustration of the behavior of Zn^2+^ electrodeposition in 3 M ZnSO_4_ and E-CS-GE.

An *in situ* optical microscope was used to record the Zn plating process in 3 M ZnSO_4_ and E-CS-GE. With increasing deposition time at a constant current of 1 mA cm^−2^, the Zn plating layer in 3 M ZnSO_4_ develops an uneven surface morphology (Fig. 3a). The side-view SEM images of the deposited Zn electrode in Fig. 3b and c show randomly distributed island-like Zn protuberances with lateral sizes of 50–100 µm, also indicated by the 3D height images (Fig. S17). A closer SEM observation reveals that the large-sized Zn protuberances are agglomerations of numerous Zn nanoflakes with no preferred orientation. Such plating behavior is believed to exacerbate a significant risk of short circuits for practical AZMBs. In contrast, with the E-CS-GE, the surface of the Zn substrate remains flat upon a 2-h Zn plating (Fig. 3d), and the thickness of the deposition layer is nearly linear to the deposition time, indicating high compactness of the deposited Zn. This is also supported by the SEM images of the plated Zn electrode from both side view and top view, and the 3D height images, where the deposited Zn layer is composed of highly regular parallel-stacked Zn plates (Fig. 3e and f, and Fig. S17). Based on the above observations and general understanding of the mechanism of dendrite growth, we reasonably deduce that the high toughness of E-CS-GE likely plays a critical role in enabling directional stacking of the Zn plates and mitigating dendritic Zn growth.

Additionally, the E-CS-GE effectively suppresses hydrogen evolution during Zn^2+^ plating, as directly observed by the reduced bubble formation in Fig. 3d, compared to the significant gas evolution in 3 M ZnSO_4_ (Fig. 3a). Linear sweep voltammetry (LSV) in a two-electrode configuration (Zn counter/reference, stainless steel working electrode) reveals an expanded electrochemical stability window of 2.31 V for E-CS-GE (Fig. S18). Post-test SEM characterization of Zn foils and Tafel analysis (Fig. S19) further confirm the enhanced corrosion resistance of E-CS-GE. Collectively, these results demonstrate the multiple functionalities of the E-CS-GE in effectively mitigating dendritic Zn growth, HER, and corrosion.

For aqueous Zn electrolytes, the Zn^2+^ solvation structure and the local environment of water are two key determinants of the electrochemical performance. Compared to liquid electrolytes, gel polymer electrolytes are distinguished by their polymer matrices actively modulating the microstructure of liquid components, thereby governing ion transport kinetics. As shown in Fig. 3g, a slight blue shift of the −NH_2_ absorption band from 1322 to 1327 cm^−1^ was observed in the Fourier transform infrared (FTIR) spectrum of the E-CS-GE compared to its salt-free counterpart (E-CS-Gel), clearly evidencing the modulation capability of the CS matrix on the Zn^2+^ solvation structure [32,33].

Low-field nuclear magnetic resonance (LF-NMR) analysis of transversal relaxation time (*T*_2_) distributions (Fig. 3h and Fig. S20) quantitatively distinguished three water states: bound H_2_O (*T*_2_ = 0.1–4 ms, tightly associated), network H_2_O (*T*_2_ = 4–50 ms, structured immobile), and free H_2_O (*T*_2_ = 50–1000 ms, bulk-like) [34]. The E-CS-GE drastically reduces free H_2_O to 5% (vs 68% in 3 M ZnSO_4_), while increasing network H_2_O to 63% (vs 1.1%), is attributed to H-bonding between H_2_O and −NH_2_ and −OH groups of CS. The O−H stretching band in the Raman spectra (Fig. 3i) also indicates that the E-CS-GE exhibits a reduced area percentage of the free water-associated component peak (centered at 3443 cm⁻¹). This attenuation is primarily attributed to the H-bonding interactions between H_2_O and −OH/−NH₂ groups on CS, with a possible contribution from the nanoconfinement effect of the CS matrix [18,35], which endowed the E-CS-GE with enhanced freezing tolerance as confirmed by differential scanning calorimetry (DSC) tests (Fig. S21).

Molecular dynamic (MD) simulation provides further insight into the H-bonds in the E-CS-GE (Table S6). It is revealed that −NH_2_ and −OH groups on CS chains form intensive H-bonds with H_2_O molecules, increasing the total number of H-bonds in the E-CS-EG while suppressing the H-bonding interactions between H_2_O molecules (Fig. S22). Additionally, the H-bonds between water molecules exhibited both a shorter lifetime and lower energy than those between CS and H_2_O molecules, indicating the stronger interactions between CS and H_2_O molecules (Fig. S23a and b). The lower number of free H_2_O molecules in the E-CS-GE (Fig. S23c) is consistent with the LF-NMR results. Additionally, the H_2_O diffusion coefficient drops from 5.74 × 10^−10^ to 5.68 × 10^−11^ m^2^ s^−1^ when the liquid components are immobilized by the CS matrix (Fig. S23d). These findings collectively demonstrate that the strong interaction between CS and H_2_O significantly suppresses water reactivity and thereby mitigates side reactions such as hydrogen evolution.

Calculated radial distribution functions (RDFs) and cumulative coordination numbers (CNs) for 3 M ZnSO_4_ and E-CS-GE are shown in Fig. 3j and k, and Fig. S24. It is revealed that the CN of H_2_O molecules around Zn^2+^ in the E-CS-GE is reduced to 4.05 (vs 4.34 for 3 M ZnSO_4_) due to the occurrence of Zn^2+^-CS coordination (mainly with −OH groups). Density functional theory (DFT) calculation results (Fig. S25) show a higher binding energy of −0.76 eV between Zn^2+^ and −OH groups on CS chains than −0.62 eV between Zn^2+^ and H_2_O. The desolvation energies of the Zn^2+^ solvate mediated by CS (represented by CS-[Zn(H_2_O)_5_]^2+^) and Zn^2+^ dominantly coordinated by H_2_O molecules of [Zn(H_2_O)_6_]^2+^ are determined to be −2.89 and −2.17 eV, respectively (Fig. 3l and Fig. S25), suggesting the enhanced Zn^2+^ desolvation kinetics in the E-CS-EG compared to its liquid counterpart.

Based on the experimental and simulation data, Fig. 3m summarizes the electrochemical deposition mechanism of Zn ions in 3 M ZnSO_4_ and E-CS-GE. In detail, (1) the coordination interaction between Zn^2+^/H_2_O and free −NH_2_/−OH groups in CS chains modulates the solvation structure and facilitates the Zn^2+^ desolvation process in the E-CS-GE; (2) during the nucleation process, the affinity of the dense CS polymer network can regulate the Zn^2+^ flow and inhibit the lateral movement of Zn^2+^ ions, thus leading to a more uniform local current distribution and homogeneous Zn deposition; and (3) the presence of abundant hydrophilic functional groups contributes to the immobilization of H_2_O molecules by the H-bonds, which in turn locks in a significant amount of free water and mitigates HER. These findings confirm the importance of the E-CS-GE in realizing uniform and controlled Zn migration and deposition, indicating its significant potential implications for the development of high-performance AZMBs.

Impressively, Zn||Zn symmetric cells using other biomacromolecular E-GEs (E-CMC-GE, E-SA-GE, and E-SF-GE) also delivered an improved cycling life of >500 h, likely owing to the optimized solvation structure and suppression of HER by the functional groups of the biopolymers. These results highlight the universality of our electrogelation strategy for constructing mechanically robust and ionically conductive biomacromolecular hydrogel electrolytes for long-life dendrite-free AZMBs (Figs S26 and S27).

### Evaluation of electrochemical performance of the Zn||ZVO cells across a wide temperature range

**Figure 4.**
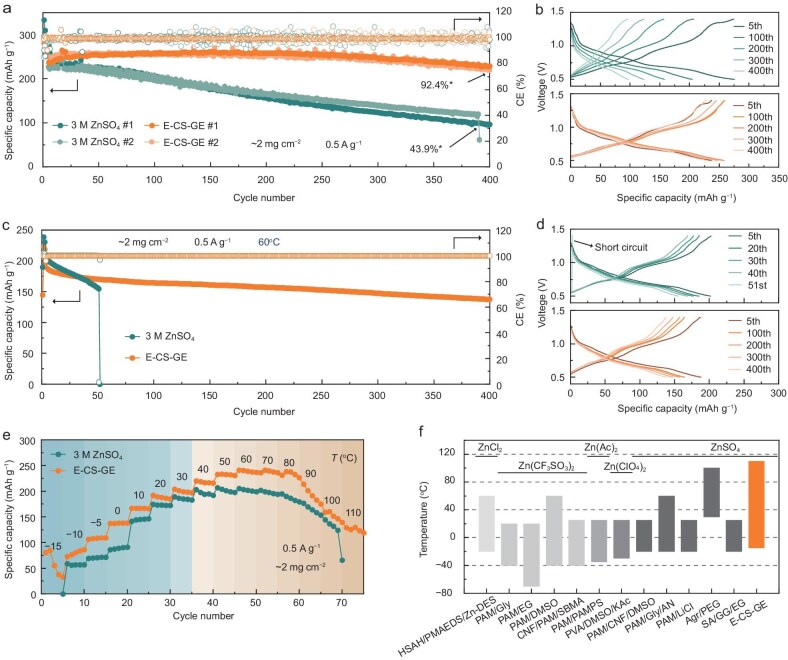
Electrochemical performance of Zn||ZVO cells with 3 M ZnSO_4_ and E-CS-GE. (a and b) Long-term cycling stability (a) and corresponding charge-discharge voltage profiles (b) at specific cycles for Zn||ZVO cells. Asterisks indicate average values. (c and d) Cycling performance (c) and corresponding charge-discharge voltage profiles (d) at specific cycles for Zn||ZVO cells at 60°C. (e) Temperature-dependent cyclability of Zn||ZVO cells from −15 to 110°C. (f) Comparison of operating temperature range of representative electrolytes targeted at wide-temperature applications.

Building on the advantages of the *in situ* E-CS-GE for the Zn anode, Zn||Zn_0.25_V_2_O_5_·nH_2_O (ZVO) cells were assembled for further investigation of the multiple merits of the E-CS-GE. The long-term cycling performance at a current density of 0.5 A g^−1^ was studied in Zn||ZVO cells using different electrolytes at 30°C. As revealed by the cycling results in Fig. 4a, the E-CS-GE exhibits an initial discharge capacity of 243.2 mAh g^−1^ (the capacity represents the average values of the fifth cycle of cell #1 and #2 unless otherwise noted) and maintains 224.7 mAh g^−1^ after 400 charge/discharge cycles, corresponding to a high-capacity retention ratio of 92.4%. In sharp contrast, the Zn||ZVO cells with 3 M ZnSO_4_ exhibit a severe capacity decay of 43.9% before the 400th cycle. Figure 4b exhibits the evolution of the charge/discharge curves of Zn||ZVO cells with different electrolytes. For the cell with the 3 M ZnSO_4_ electrolyte, the specific capacity decreases continuously upon cycling along with voltage decay, whereas with the use of the E-CS-GE nearly overlapped charge/discharge voltage curves are observed, demonstrating the superior reversibility of Zn^2+^ intercalation chemistry in the ZVO cathode enabled by the E-CS-GE due to its ability to stabilize the interface against mechanical/chemical degradation (Figs S28 and S29). More remarkably, the Zn||ZVO cell with E-CS-GE delivers high specific capacities from 266 to 33 mAh g^−1^ at current densities from 0.1 to 5.0 A g^−1^. When the current density returns to 1.0 A g^−1^, it recovers 100% of the initial capacity (205 mAh g^−1^). These results demonstrate the outstanding high-rate capability of E-CS-GE in Zn||ZVO cells (Fig. S30).

For aqueous electrolytes whether in liquid or gel form, their application at elevated temperatures is highly challenging due to the enhanced intrinsic reactivity of water and the increased thermodynamic instability between water and metallic Zn. At 60°C in Fig. 4c and d, the cell with the 3 M ZnSO_4_ quickly fails after 51 cycles due to short circuit. With the E-CS-GE, the cycle life achieves >400 cycles with a specific capacity of 154.5 mAh g^−1^ and a capacity retention ratio of 73.2%. To explore the practical operating temperature limits of the E-CS-GE, the cyclability of Zn||ZVO cells was investigated from lower to higher temperatures (Fig. 4e). The cell with 3 M ZnSO_4_ is inoperable at −15°C and suffers from electrical failure beyond 100°C. E-CS-GE further widens the low and high operating temperature limits to −15 and 110°C, with higher discharge specific capacities than 3 M ZnSO_4_ across the whole temperature range, showing its ability to offer faster reaction kinetics, especially at subzero temperatures. Compared with 3 M ZnSO_4_, thermal analysis further reveals that the E-CS-GE exhibits a lower freezing point with retarded crystallization kinetics, and higher thermal stability (Figs S21 and S31). Additionally, the reduced free water content in E-CS-GE enhances thermal stability, as suggested by the LSV results obtained at 100°C (Fig. S32). These two benefits jointly lead to the impressively high upper limit of the operating temperature of E-CS-GE.

Combined with the aforementioned findings, Zn||ZVO cells with the E-CS-GE show no obvious deterioration in cycling performance across a wide operating temperature range, demonstrating their good capability in the practical operating temperature range from −15 to 110°C. Compared with state-of-the-art aqueous electrolytes (Fig. 4f and Table S7) [8,19,36–45], the E-CS-GE exhibits a wider operating temperature window (−15 to 110°C). Consequently, our work successfully improves the operating temperature range of AZMBs without introducing organic additives and co-solvents, thus broadening the potential application of these energy storage devices.

### Long-term cycling performance of full cells under practical conditions

**Figure 5.**
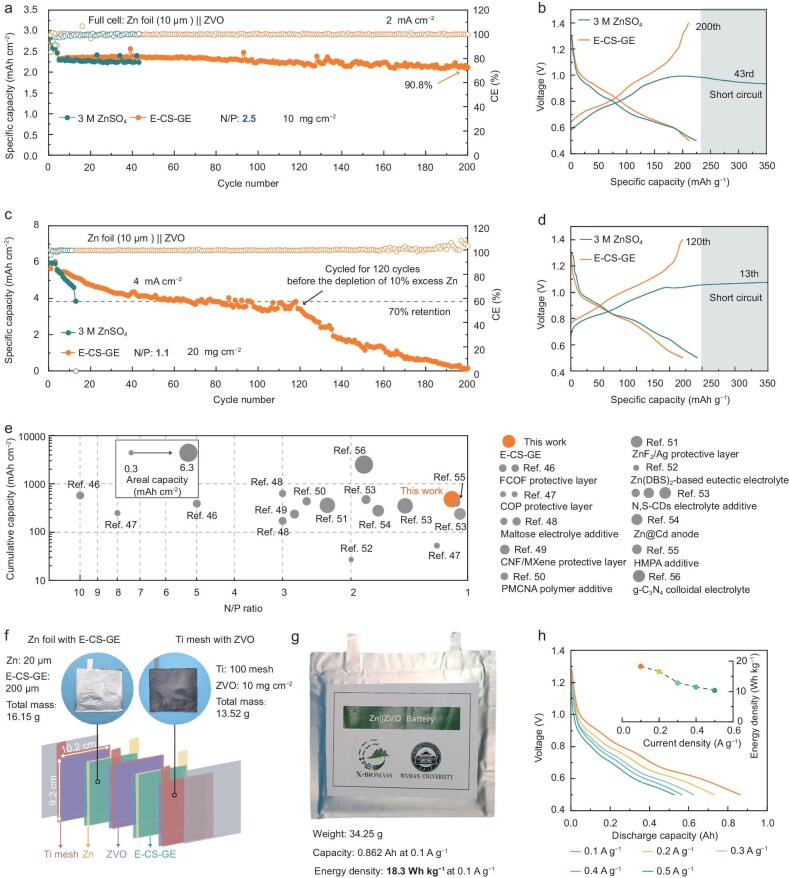
Electrochemical cycling performance of practical full cells. (a) Long-term cycling stability of Zn||ZVO full cells at an N/P ratio of 2.5. (b) Charge-discharge voltage profiles at the 43rd cycle of the Zn||ZVO cell with 3 M ZnSO_4_ and the 200th cycle of the Zn||ZVO cell with E-CS-GE. (c) Cycling performance of Zn||ZVO cells with a reduced N/P ratio of 1.1. (d) Galvanostatic charge-discharge profiles of the full cells at the 13th cycle (3 M ZnSO_4_) and the 120th cycle (E-CS-GE). (e) Comparison of N/P ratio and cumulative capacities between this work and previously reported Zn-based full cells. (f) Schematic of the multilayered structure of a Zn||ZVO pouch cell. Insets: Zn foil coated with E-CS-GE and Ti mesh loaded with ZVO. (g) Photograph of the fabricated Zn||ZVO pouch cell employing the E-CS-GE. (h) Discharge profiles of the pouch cell at current densities ranging from 0.1 to 0.5 A g^−1^. Inset: energy density versus current density.

The striking performance of the Zn||ZVO half-cell achieved by the E-CS-GE encourages further investigation of the electrolyte's practicality under constrained conditions of high-areal-capacity cathodes (>2 mAh cm^−2^) and low N/P ratios. By combining a 10-μm-thick Zn foil anode and a high-mass-loading ZVO cathode (10 mg cm^−2^), the N/P ratio of a Zn||ZVO cell is controlled to be ∼2.5. Under such a condition, the cell cycled with 3 M ZnSO_4_ can operate for merely 43 cycles before short-circuiting, while a significantly enhanced cyclability is achieved with the cell based on the E-CS-GE, showing a remarkably high capacity retention ratio of 90.8% over 200 cycles (the initial formation cycles were not counted, Fig. 5a and b).

By further reducing the N/P ratio to a commercial-level of 1.1 using a 20 mg cm^−2^ ZVO cathode (Fig. 5c and d), it is observed that the specific areal capacity of the Zn||ZVO cell with 3 M ZnSO_4_ monotonically decreased markedly within the very limited cycling lifetime of 13 cycles due to the exhaustion of cyclable Zn. This is rather different from the above case in which a 1.5-time excess Zn was provided. The cell with the E-CS-GE maintains stable operation for 120 cycles with a gradual capacity fade, showing a respectable capacity retention of ∼70%. The cumulative discharge capacity of ∼500 mAh cm^−2^ for a full cell achieved at a N/P ratio of 1.1 represents one of the most striking results among various aqueous AZMB chemistries ever reported (Fig. 5e and Table S8) [46–56]. This result is attainable only when the Zn cycling process is highly reversible, highlighting the potential of our E-GE in promoting practical AZMBs.

To assess the industrial feasibility of the E-CS-GE, we constructed Ah-level pouch-type cells with configuration and parameters as shown in Fig. 5f and g, and Table S9. The pouch-type cell initially delivers a discharge capacity of 862 mAh at 0.1 A g^−1^ (4 mA cm^−2^), corresponding to a specific energy of 18.3 Wh kg^−1^ based on the total mass of the pouch cell, and retains 57% of its specific energy from 0.1 to 0.5 A g^−1^ (Fig. 5h). It is worth noting that, despite these achievements, further optimization of cell configuration, cell parameters, and test conditions is needed to achieve higher energy density and reasonable cyclability for real-world applications.

### Sustainability of the E-CS-GE with environmental and economic benefits

**Figure 6.**
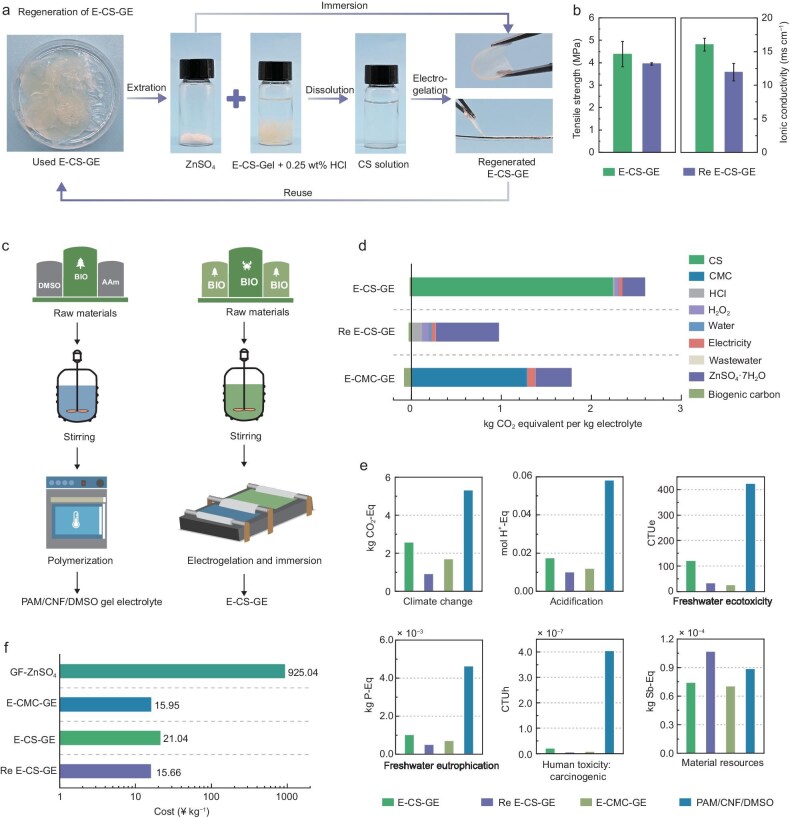
Recyclable E-CS-GE with environmental and economic benefits. (a) The recycling process of the E-CS-GE. (b) Comparison of the tensile strength and ionic conductivity of the E-CS-GE before and after recycling. (c) Comparison of manufacturing routes of the PAM/CNF/DMSO hydrogel electrolyte (left) to E-CS-GE (right). (d) Disaggregated climate change impact for E-CS-GE, Re E-CS-GE, and E-CMC-GE (a dynamic accounting for biogenic carbon storage is applied with a 20-year lifespan). (e) Environmental impacts of E-CS-GE, Re E-CS-GE, and E-CMC-GE in six relevant categories when compared against petroleum-based GPE. (f) Comparison of the economic costs of E-CS-GE, Re E-CS-GE, and E-CMC-GE with the traditional GF separator with 3 M ZnSO_4_ (horizontal axis: log scale).

From the perspective of resource sustainability and environmental protection, recycling is a necessary process to manage end-of-life batteries. We confirmed the recyclability of the E-CS-GE via a facile route as shown in Fig. 6a. The used E-CS-GE was first soaked in water to allow for the dissolution of ZnSO_4_. White powders (ZnSO_4_) were obtained after evaporating the resulting solution. The salt-free CS hydrogel was dissolved again in a 0.25 wt% HCl solution for a secondary electrogelation to obtain the regenerated E-CS-GE (named Re E-CS-GE). The measured tensile strength and ionic conductivity of the Re E-CS-GE are quite close to those of the virgin E-CS-GE sample (3.95 vs 4.40 MPa (Fig. 6b and Fig. S33) and 11.9 vs 16.0 mS cm^−1^ (Fig. 6b and Fig. S34)), suggesting the excellent recyclability and reusability of electrogelated biomacromolecular hydrogel electrolytes.

In this context, we assessed the environmental sustainability of the electrogelated biomacromolecular hydrogel electrolytes through life cycle assessment (LCA), a well-established methodology for determining environmental impacts [57,58], including an extensively studied PAM-based electrolyte as the control for comparison (Fig. 6c). The disaggregated climate change potential resulting from the production of E-CS-GE, Re E-CS-GE, and E-CMC-GE (*cradle-to-gate*) is shown in Fig. 6d and e (*Environmental Footprint v 3.1* LCIA methodology, further details in Fig. S35 and Tables S10–S14). The greenhouse gas emissions for the production of the E-CS-GE and E-CMC-GE are estimated to be 2.58 and 1.70 kg CO_2_ equiv·kg^−1^, respectively. In contrast, the *cradle-to-gate* climate change of PAM/CNF/DMSO [8] electrolytes utilizing the same renewable electricity mix (life cycle inventory from secondary sources, see Fig. S35e and Tables S15 and S16) was found to be 5.33 kg CO_2_ equiv·kg^−1^. A limited effect of biogenic carbon credits is observed due to the dynamic accounting approach used, which in an analogy to monetary commodities, applies a discount rate based on the operating lifespan of the material [59,60]. Since the lifespan of the material is considered to be 20 years, a fifth of the total embedded biogenic carbon is considered for crediting purposes. Very importantly, the E-CS-GE can be recycled using the procedure shown in Fig. S35c and Table S11, and be utilized again in an AZMB at a reduced carbon footprint of 0.94 kg CO_2_ equiv·kg^−1^ (the credits from biogenic carbon are increased due to the higher concentration of CS in the electrolyte). During recycling, ZnSO_4_ use presents the largest burden with a share of 71.6%. Nonetheless, the Re E-CS-GE not only displays reduced environmental impacts but also holds key attributes to close material loops through simple and green processes.

To provide a more complete overview of the environmental sustainability of the electrolytes, we also analyzed other relevant environmental impact categories (Fig. 6e and Table S16). Our E-CS-GE, and especially the Re E-CS-GE, not only offers a reduced CO_2_ footprint compared to non-renewable electrolytes (PAM/CNF/DMSO), but also presents significantly lower impacts in relevant impact categories including *acidification, freshwater ecotoxicity, eutrophication* (*freshwater/marine/terrestrial*), *human toxicity carcinogenic*, and *material resources*. Furthermore, the obtained impact values could be significantly reduced in the near future as production is upscaled by methods such as roll-to-roll, which optimize material and energy use. This is of particular relevance for the future sustainable energy landscape, where material manufacturing is considered to be a major driver of environmental impacts [61]. Such an environmentally friendly nature of our biomacromolecular hydrogel electrolytes is attributed to the use of renewable materials, the avoidance of toxic solvents, the energy-efficient fabrication process, and their easy recyclability.

Furthermore, it should be noted that the global market for GPEs is projected to grow at a compound annual growth rate (CAGR) of 12.9% between 2022 and 2031, reflecting its increasing importance in energy storage (https://www.transparencymarketresearch.com/gel-polymer-electrolytes-market.html). To that end, in order to ensure the practical implementation of our proposed technology in sustainable batteries, we conducted a techno-economic assessment (TEA) for our developed biomacromolecular hydrogel electrolytes (see Table S17 for a detailed account of the assumptions). As shown in Fig. 6f and Table S18, the production cost of the E-CS-GE, Re E-CS-GE, and E-CMC-GE is 21.04 ¥ kg^−1^ (2.90 $ kg^−1^ based on the exchange rate as of July 2024), 15.66 ¥ kg^−1^ (2.14 $ kg^−1^), and 15.95 ¥ kg^−1^ (2.20 $ kg^−1^), respectively, which are all much lower than that of the traditional glass fiber (GF) separator with ZnSO_4_ electrolyte (925.04 ¥ kg^−1^/127.42 $ kg^−1^). Notably, when compared to conventional GPEs based on petroleum resources (polyethylene oxide, polyvinylidene fluoride, PAM), which are currently applied at the forefront of gel electrolyte research, our biomacromolecular hydrogel electrolytes still show clear competitiveness (2.14–2.90 $ kg^−1^ vs potential values of 100–300 $ kg^−1^ for PAM-based electrolyte, which includes 10–20 $ kg^−1^ for the polymer matrix, 50–100 $ kg^−1^ for salts, 5–10 $ kg^−1^ for solvents, 10–50 $ kg^−1^ for additives, plus manufacturing costs) [62,63]. Taken together, the LCA and TEA results demonstrate the environmental and economic advantages of the biomacromolecular hydrogel electrolytes developed here, which balance reduced impacts in comparison to competitors while maintaining economic feasibility.

## CONCLUSION

In summary, we propose a facile yet effective electrogelation strategy with a well-controlled gelation process (thickness, structure, composition, surface adaptability, etc.) to fabricate biomacromolecular hydrogel electrolytes from a variety of renewable biomass (CS, CMC, SA, and SF). This facile approach enables the fabrication of scalable hydrogel electrolytes with a combination of superior mechanical performance and high ionic conductivity. As a proof-of-concept demonstration, the E-CS-GE effectively inhibits the uncontrolled formation of Zn dendrites and the side reaction, owing to the functional groups of CS, excellent mechanical properties and sufficient ionic conductivity. The application of E-CS-GE guarantees a long cycling lifespan (4500 h at 1 mA cm^−2^ and 1 mAh cm^−2^), high Zn utilization (300 h with DOD of 80%), and exceptional plating/stripping reversibility (an average CE of 99.83% over 2800 cycles) for Zn cycles. In addition, the practicability of the E-CS-GE is demonstrated by good cycling stability even at a low N/P ratio of 1.1 and a wide operating temperature range from −15 to 110°C, suitable for practical applications under extreme environments across cold and hot climates. Moreover, the biomacromolecular hydrogel electrolytes show outstanding recyclability, low environmental impacts, and high economic feasibility. Such excellent environmental and economic sustainability, combined with their outstanding performance under practical and extreme conditions, offers unlimited possibilities for the development of sustainable batteries.

## METHODS

Details of the materials and methods are available in the online supplementary file.

## Supplementary Material

nwaf308_Supplemental_File
