# Supplementary material for: Practical, sustainable, wide-temperature-adaptable zinc-metal batteries enabled by electrogelated recyclable biomacromolecular hydrogel electrolytes
Source: Natl Sci Rev. 2025 Jul 31;12(9):nwaf308. doi: 10.1093/nsr/nwaf308 (PMC12418938; doi:10.1093/nsr/nwaf308)
Supplement: nwaf308_Supplemental_File [file nwaf308_supplemental_file.pdf]

Supplementary Information

Practical, sustainable, wide-temperature-adaptable zinc-metal batteries enabled by electrogelated recyclable biomacromolecular hydrogel electrolytes

Jing Huang[1,†], Sijun Wang[1,†], Le Yu[1,*], Erlantz Lizundia[2], Xuanyu Zeng[1], Lu Chen[1], Ziyang Lu[1], Luhe Qi[1], Yu Lin[1], Hongbing Deng[1], Chaoji Chen[1,*]

1. Hubei Biomass-Resource Chemistry and Environmental Biotechnology Key Laboratory, Hubei Provincial Engineering Research Center of Emerging Functional Coating Materials, School of Resource and Environmental Sciences, Wuhan University, Wuhan 430079, China;
2. Life Cycle Thinking Group, Department of Graphic Design and Engineering Projects. University of the Basque Country (UPV/EHU), Bilbao 48013, Spain;

***Corresponding authors**. E-mails: yuleee@whu.edu.cn; chenchaojili@whu.edu.cn

[†] Equally contributed to this work.

Supplementary Methods

Materials:

Chitosan (CS, 200–600 mPa·s, Deacetylation: 85–90%) was purchased from Tokyo Chemical Industry Co., Ltd. (Japan). Sodium alginate (SA) and carboxymethyl cellulose (CMC) were purchased from Shanghai Macklin Biochemical Co., Ltd. (China). Silk fibroin (SF) was extracted from *Bombyx mori* cocoons (Cellmatrix Co., Ltd., China). Bacterial cellulose nanofiber was purchased from Guilin Qihong Technology Co., Ltd. (Guilin, China). Hydrochloric acid (HCl, 37 wt%), hydrogen peroxide (H_2O_2, 30 wt%), $Al_2(SO_4)_3 \cdot 18H_2O$, $CaCO_3$, and methanol were purchased from Sinopharm Chemical Reagent Co. Ltd., Shanghai, China. $ZnSO_4 \cdot 7H_2O$ was supplied by Aladdin Chemical Reagent Co. Ltd. Zinc plates with a thickness of 100 and 10 μm were obtained from commercial resources in China. All other chemicals were of analytical grade and were used as received.

Preparation of E-GE: E-CS-GE: CS powder was slowly added to an aqueous solution of 0.15 wt% HCl under magnetic stirring at room temperature to obtain the CS solutions with certain concentrations. 0.2 g of 30 wt% H_2O_2 solution was added to 100 g CS solution to inhibit the generation of hydrogen bubbles during the electrolysis and generate a pH gradient at a lower reducing voltage [1]. A Zn plate and a graphite electrode were immersed in the CS solution as the cathode and the anode, and then a constant current density of 0.5 mA cm^{-2} was applied to obtain the E-CS-Gel. The CS gel was fully washed with deionized water, followed by immersion in a 3 M $ZnSO_4$ aqueous electrolyte to obtain the E-CS-GE.

E-CMC-GE: A 1 wt% CMC solution was prepared by dissolving CMC powder in deionized water under continuous magnetic stirring at room temperature until a homogeneous solution was achieved. A Zn plate and a graphite electrode were immersed into the CMC solution as the anode

and cathode, respectively. A constant current density of 0.5 mA cm^{-2} was applied to obtain the E-CMC-Gel. The resulting E-CMC-Gel was rinsed with deionized water to remove residual unreacted species, followed by immersion in 1 wt% $Al_2(SO_4)_3$ solution. The gel was then rinsed briefly with deionized water to eliminate excess Al^{3+} ions and subsequently soaked in 3 M $ZnSO_4$ electrolyte to obtain the E-CMC-GE.

E-SA-GE: SA powder and $CaCO_3$ were dissolved in deionized water under magnetic stirring at room temperature to prepare a homogeneous solution containing 1 wt% SA and 0.25 wt% $CaCO_3$. Electrochemical gelation was performed by immersing a Zn plate (anode) and a graphite electrode (cathode) into the SA/$CaCO_3$ solution, followed by applying a constant current density of 0.5 mA cm^{-2} to form the E-SA-Gel. The E-SA-Gel was rinsed with deionized water and subsequently immersed in 3 M $ZnSO_4$ electrolyte to obtain the final E-SA-GE.

E-SF-GE: Pure SF was extracted from *Bombyx mori* cocoons (Cellmatrix Co., Ltd., China) using a modified degumming method [2]. Briefly, 10 g of cocoons were boiled in 4 L of 0.02 M Na_2CO_3 solution for 30 min. The degummed SF fibers were rinsed thoroughly with deionized water, air-dried, and then dissolved in 9.3 M LiBr solution at 60 °C for 4 h, followed by dialysis (Beyotime dialysis tubing, 3500 MWCO) for 4 days to remove LiBr. The pure SF solution was freeze-dried and stored for further use. An 8 wt% SF solution was prepared by dissolving the freeze-dried SF in deionized water under gentle magnetic stirring. The fresh solution was aged at 60°C for 8 h. Next, a Zn plate (anode) and a platinum plate (cathode) were immersed into the aged SF solution, and a direct current voltage of 25 V was applied to induce SF gelation. The resulting SF hydrogel was rinsed with deionized water to remove unbound SF. To stabilize the structure, the SF hydrogel was immersed in 70 wt% methanol solution. After rinsing again with deionized water, the SF hydrogel was soaked in 3 M $ZnSO_4$ solution to obtain E-SF-GE.

Recycling of Re E-CS-GE: Waste E-CS-GE was immersed in deionized water to remove $ZnSO_4$. The mixture was filtered to separate the E-CS-Gel from $ZnSO_4$ solution. The filtrate ($ZnSO_4$ solution) was heated to evaporate water until a crystal film formed on the surface. The solution was then cooled down to precipitate $ZnSO_4 \cdot nH_2O$ as white crystals. The $ZnSO_4$-depleted E-CS-Gel was dissolved in a 0.25 wt% HCl solution under vigorous stirring to obtain a homogeneous 1 wt% CS solution. Subsequently, the regenerated CS solution was used to prepare Re E-CS-GE following the identical procedure as described for the original E-CS-GE.

The liquid 3 M $ZnSO_4$ electrolyte was adopted as the control to compare the electrochemical performance.

Preparation of ZVO cathode: ZVO was prepared via a modified hydrothermal method based on prior work [3]. Typically, 0.54 g V_2O_5 and 0.215 g $Zn(OAc)_2$ were dissolved in a solvent mixture containing 70 mL deionized water, 2 mL 10 wt% HNO_3, and 5 mL acetone. The mixture was transferred to a Teflon-lined stainless-steel autoclave and heated in an oven at 180°C for 24 h. After been allowed to cooling to room temperature naturally, the resulting precipitate was collected by vacuum filtration, washed sequentially with deionized water and isopropanol to remove impurities, and dried in an oven at 80°C to remove the free water. The cathode was prepared using a vacuum infiltration method. An aqueous suspension was formulated by homogeneously mixing ZVO, bacterial cellulose (BC) nanofiber, and carbon nanotube in a 75:20:5 weight ratio in deionized water. The mixture was sonicated to ensure dispersion and vacuum-infiltrated into a stainless-steel mesh current collector. The electrode was dried at 60°C for 6 h to remove residual water. Unless otherwise specified, the ZVO mass loading was approximately 2 mg cm^{-2}.

Characterizations:

The morphologies of all samples were characterized using field emission scanning electron microscopy (FE-SEM, Zeiss, GeminiSEM 500, Germany), a 3D optical profiler (New View 9000, USA), and an optical microscope (Sunny, China). Digital photos of the E-GE were acquired using a Canon camera. Elemental distribution analysis was analyzed by energy-dispersive X-ray spectroscopy (EDS) coupled with FE-SEM. Fourier transform infrared (FTIR) spectra of the E-CS-Gel and E-CS-GE were recorded on a Thermo Scientific Nicolet FTIR 5700 spectrometer (USA). Thermogravimetric analysis (TGA) and differential scanning calorimetry (DSC) analysis (Mettler-Toledo, TGA2/DSC3, Swiss) tests were carried out under an air atmosphere, with temperature ramping from −80 to 200°C at a rate of 10°C min^{-1}. Low-field nuclear magnetic resonance (LF-NMR) analyzer (Niumag, MesoMR23-060H-I, China) was used to test the water distribution of the electrolytes. The mechanical properties of the E-GE were evaluated via tensile testing using a universal testing machine (Shimadzu, AGS-X, Japan).

Electrochemical measurements:

Electrochemical tests were performed on Zn||Zn, Zn||Cu, and Zn||ZVO assembled in CR2032 coin-type cells in an ambient environment using a LAND CT2003A battery testing system at 30°C. Zn||Zn symmetric cells were assembled by two Zn foils (100 μm, unless otherwise specified), separated by the E-GE or GF separator with 3 M $ZnSO_4$ liquid electrolyte. Zn||Cu asymmetric cells were fabricated using Cu foil (working electrode) and Zn foil (reference and counter electrode). Zn||ZVO cells were assembled with Zn foil (100 μm, unless otherwise specified) as the anode and ZVO (around 2 mg cm^{-2}, unless otherwise specified) as the cathode. Zn||ZVO full cells were

assembled with 10 μm Zn foil and ZVO cathodes at 10 or 20 mg cm^{-2} to achieve an N/P ratio of 2.5 or 1.1, respectively, where the N/P is the ratio of negative capacity to positive capacity.

The cumulative capacity (Q) was calculated based on the following Equation (1):

$$Q = i \times t \tag{1}$$

where Q (mAh) represents the cumulative capacity, i (mA) represents the current, and t (h) denotes charge/discharge duration.

DOD for Zn metal anodes was calculated according to the following Equation (2):

$$DOD = \frac{C_r}{C_t} \times 100\% \tag{2}$$

where Cr (mAh) represents the capacity involved in the electrode reaction, and Ct (mAh) is the theoretical total capacity of the Zn anode.

The ionic conductivity (σ, mS cm^{-1}) of E-GE was determined via electrochemical impedance spectroscopy (EIS) using symmetric stainless steel||stainless steel cells. Measurements were conducted on a CHI760E electrochemical workstation (Chenhua Instruments, China). The ionic conductivity was calculated according to the following Equation (3):

$$\sigma = \frac{L}{RS} \tag{3}$$

where L (cm) is the thickness of the E-GE, R (Ω) is the bulk resistance derived from the high-frequency intercept of the Nyquist plot on the real impedance axis, and S (cm^2) is the effective contact area between the stainless-steel electrode and electrolyte.

The Zn^{2+} transference number (t_+) was determined for Zn||Zn cells with the E-CS-GE using the Bruce-Vincent method, with a control group consisting of GF separators soaked in 3 M $ZnSO_4$ electrolyte. Briefly, a direct-current polarization potential (ΔV) of 10 mV was applied until the current stabilized. EIS spectra were recorded before and after polarization. The Zn^{2+} transference number was calculated using Equation (4):

$$t_{+} = \frac{I_{SS}\times(\Delta V - I_0\times R_0)}{I_0\times(\Delta V - I_{SS}\times R_{SS})} \quad (4)$$

where t_{+} is the transference number, I_0 and I_{ss} are the initial and steady-state currents (mA), R_0 and R_{ss} are the initial and steady-state interface resistances (Ω), and ΔV is the applied polarization potential (10 mV).

Binding energy between molecule clusters:

The Gaussian 09 package was used to calculate the LUMO, HOMO, and binding energy of the molecule clusters. All structures were optimized considering solvent effects using the SMD model for water. The structures of molecules were optimized with B3LYP/6-311+G(d) for C, O, N, H, and S elements, and B3LYP/SDD for the Zn element. Vibrational frequency calculations at the same level of theory were performed on all optimized structures to confirm stationary points as minima [4]. The binding energy between two components (A, B) is defined as Equation (5):

$$E_b = E_{AB} - E_A - E_B - E_{BSSE} \quad (5)$$

where E_A, E_B, and E_{AB} are the total energies of A, B, and AB complexes, respectively. E_{BSSE} is the basis set superposition error correction energy, used for correcting the geometries and interaction energies [5]. The O–H bond orders were acquired based on the Mayer bond order by further calculation of Gaussian check files on Multiwfn [6].

Molecular Dynamics (MD) simulations:

MD simulations for the electrolyte structures, including the solvation structure of Zn^{2+} and water–polymer complexes, were investigated by GROMACS package with the General Amber Force Field (GAFF) [7]. The MD parameters for Zn^{2+} were in the Merz force-field parameters [8]. Water molecules were simulated in the SPC/E model [9]. The MD parameters for SO_4^{2-} and natural

polymer chains were generated using ACPYPE, and the corresponding atom charges were based on the restrained electrostatic potential (RESP) charges [7]. The constant-pressure and temperature (NPT) ensembles were first performed at 298.15 K for 10 ns to ensure system equilibrium, and then another NPT run of 30 ns was used for the post-analysis. The RDFs were calculated from the built-in module in the GROMACS package. The MD simulation visualization was achieved by visual molecular dynamics (VMD). The parameters of the simulated electrolyte are listed in Table S6.

Life cycle assessment (LCA):

We quantified the *cradle-to-gate* environmental impacts of *"E-CS-GE"* and *"E-CMC-GE"* utilizing LCA and following the ISO 14040/44 international standards [10]. The system boundary included resource extraction to the factory gate, as in previous reports on biobased materials [11-13]. Specifically, raw materials and their corresponding upstream processes, the energy required for *on-site* production, and the resulting waste management were considered. The transportation of each chemical was also included in the selected inputs as a "market", typically via freight train, freight lorry, or freight ship. The emissions caused by equipment were not considered due to the large amortization times. A low-voltage 100% renewable energy grid representing the current proportion in Switzerland was considered a representative energy mix in the context of the forthcoming renewable scenario. The flowcharts and tables in the supporting information, as shown in Fig. S35a-d and Tables S10-S13, summarize the life cycle inventory (LCI) with the utilized input and output data. The modeling of CS is based on the study by *Riofrio et al.*, with minor modifications (i.e., implementation of a 100% renewable energy grid) [14]. Besides, as the chitin source utilized in this study originated from waste streams, a burden-free approach was

taken according to the cut-off allocation. The crustacean exoeskeletons were assumed to be sourced from an average distance of 150 km and were transported by freight using a 16-32 metric ton lorry (EURO5). For comparison, the environmental impacts of a representative fossil-based hydrogel electrolyte applied in zinc ion batteries were also considered (Fig. S35e and Table S15).

The utilization of biomass has the potential to contribute to net-zero emissions given its capacity for carbon sequestration. However, during modeling, the actual service life should be considered to avoid the double counting of carbon credits. Consequently, we used a dynamic accounting approach for CO_2 calculation incorporating biogenic carbon storage [15]. A discount rate was applied to biogenic carbon with a use time of twenty years, and stoichiometry (0.273 kg of carbon produces 1 kg of carbon dioxide). As such, 1 kg of CS ($C_{18}H_{35}N_3O_{13}$, 25.2 % carbon content) and CMC ($C_8H_{15}NaO_8$, 36.6 % C content) store 0.92 and 1.34 kg CO_2, respectively. The OpenLCA 2.1.0 software with the ecoinvent v3.10.1 cut-off unit was used for the calculations. The environmental impacts were obtained according to the Environmental Footprint (EF, v3.1) methodology, which adheres to the recommendations of the European Commission for the measurement of environmental performance, such as those applied in *Product Environmental Footprint* and *Organisation Environmental Footprint* [7]. To normalize the impacts, a mass-based functional unit (FU) was applied, allowing results to be obtained based on 1 kg of material.

Economic assessment:

The economics for the fabrication of *"E-CS-GE"* and *"E-CMC-GE"* in the year 2024 was conducted for an industrial plant located in Wuhan (Hubei Province, China) having a production capacity of 360 tons·year^{-1}. Table S17 presents the considered assumptions for raw material and energy costs, together with labor, property, plant, and equipment costs. The modeled plant needs

50 workers with an 8-h single work shift, and 2080 h worked annually. Labor cost includes a direct salary of 12.5 ¥ h^{-1}, with 30% for indirect costs (overhead) and 20% for fringe coefficient (benefits and additional costs). An overall equipment effectiveness of 90% is considered to account for fabrication stops due to equipment maintenance, raw material supply interruptions, or facility downtime (90‑95% is considered for biorefineries) [8, 9]. The equipment is amortized over a period of twenty years, with 6.7% of capital costs (equipment and factory interests), 5% of maintenance, 1% of insurance, 16% of offsite/outside battery limits (space costs for utility connections and lighting), 20% of engineering, and 10% to contingencies. Microsoft Excel was utilized for the calculations [16]. Overall, the plant has 10,000 m^2 of built area at a cost of 300 ¥ m^{-2} with a thirty-year amortization. Similar assumptions were considered by our group for biomass processing [17].

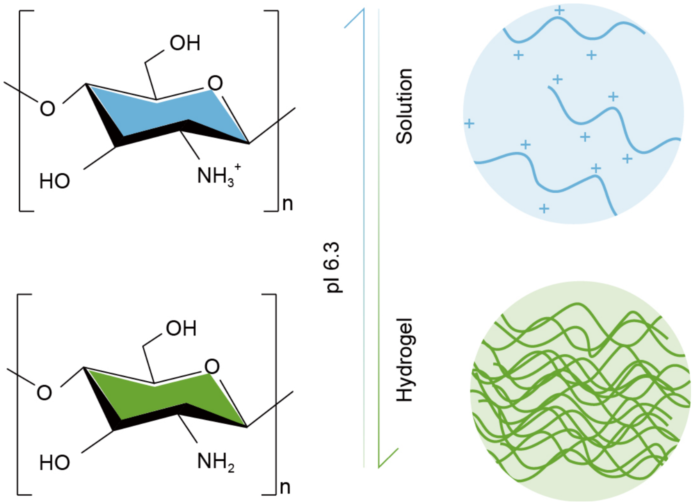

Figure S1. Schematic illustration of the pH-solubility of CS.

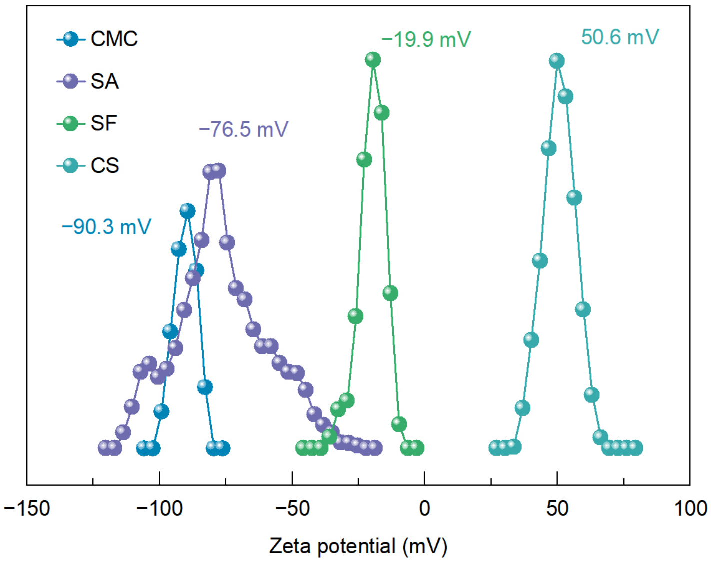

Figure S2. Zeta potential of 1 wt% CMC, 1 wt% SA, 8 wt% SF, and 1 wt% CS solution.

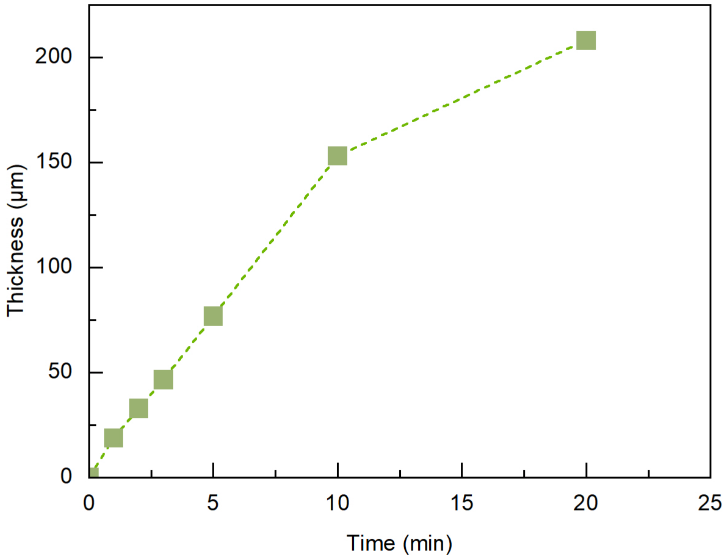

Figure S3. Thickness increase of the E-CS-Gel with electrogelation time according to the optical images.

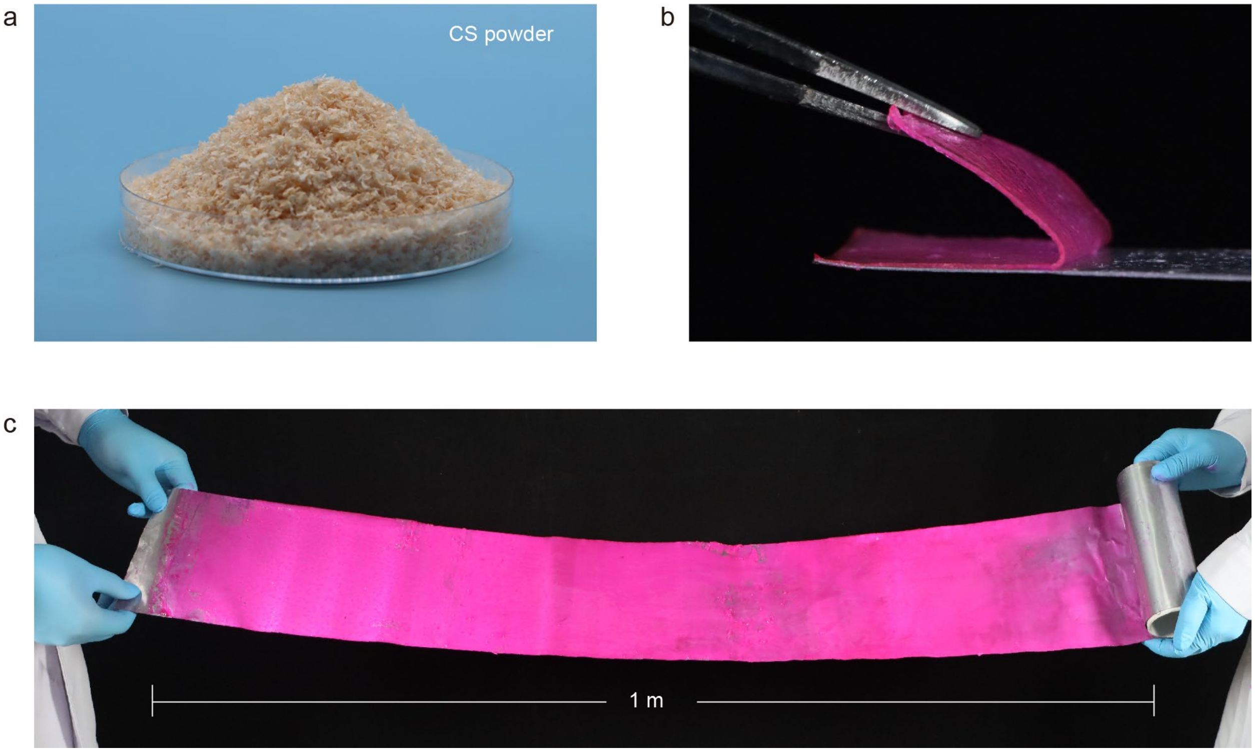

Figure S4. (a) CS powder. (b, c) Digital photographs of the E-CS-GE on a Zn foil (dyed red with rhodamine B).

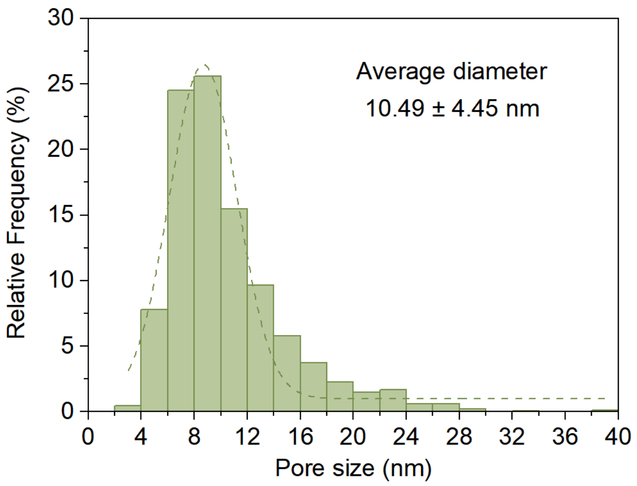

Figure S5. Pore size distribution of the E-CS-GE.

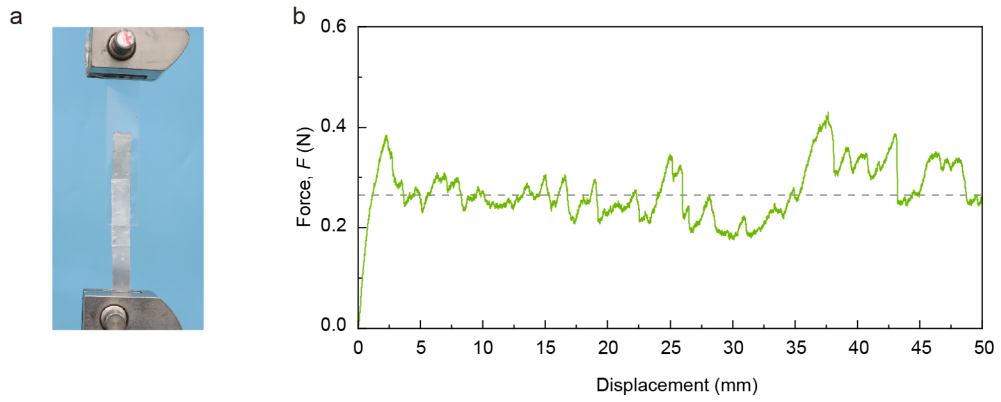

Figure S6. (a) An optical photograph of the E-CS-GE peeled off from the Zn foil. (b) 180-degree peel force-displacement curve for the E-CS-GE adhered to the Zn foil.

180-degree peeling tests were performed to evaluate the adhesion strength between the E-CS-GE and the Zn foil. The force-displacement curve reveals a high peeling force of approximately 0.27 N, indicating the strong adhesion of the electrogelated hydrogel to the Zn foil (Fig. S6). The mechanism of such in situ adhesion is straightforward: in addition to the ubiquitous hydrogen bonding interaction, the negatively charged $Zn(OH)_2$ layer formed in situ on the Zn foil during the electrogelation process ($Zn + H_2O_2 + 2e^- \rightarrow Zn(OH)_2$) strongly binds with the positively charged CS chains [18]. This advantageous characteristic for battery assembly fundamentally distinguishes the electrogelated hydrogels from conventional solution-cast hydrogels.

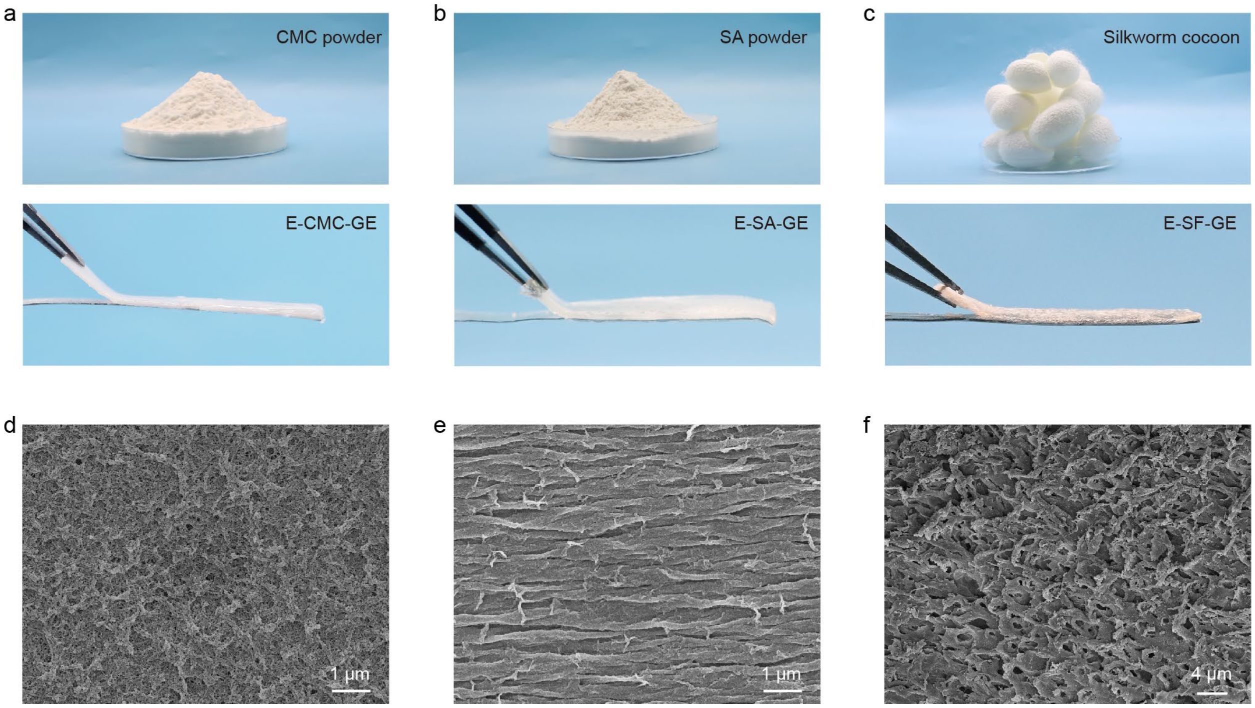

Figure S7. Expansion of biomacromolecules to prepare (a) E-CMC-GE, (b) E-SA-GE, and (c) E-SF-GE for evaluating the universality of the E-GE strategy. SEM images of the (d) E-CMC-GE, (e) E-SA-GE, and (f) E-SF-GE.

We further explored the applicability of this *in situ* electrogelation strategy to different kinds of biomacromolecules to demonstrate its universality. It was found that some other charged biomacromolecules such as CMC, SA, and SF can also form hydrogels adhering to the Zn metal surface (Fig. S7), with the underlying microscopic mechanism described as follows: (1) Under a certain electric field, the negative CMC molecules (−90.3 mV, Fig. S2) migrate to the Zn electrode, and Zn^{2+} generated from the Zn plate can coordinate with the abundant carboxyl groups and hydroxyl groups of CMC molecules to form a stable E-CMC-GE on the Zn electrode surface [19]. (2) The E-SA-GE was obtained from the SA solution (−76.5 mV, Fig. S2), containing dispersed $CaCO_3$ particles. The H^+ generated at the anode surface resulted in the dissolution of $CaCO_3$ to produce Ca^{2+}. The locally released Ca^{2+} interacts with SA macromolecules to form a Ca-crosslinked hydrogel network [20]. (3) For the SF solution (−19.9 mV, Fig. S2), electrical stimuli promote the migration of negatively charged SF molecules toward the Zn electrode, and the oxidation of H_2O generates H^+ resulting in the reduction of pH around the Zn electrolyte. When

the pH is below 4.8, the aggregated SF molecules self-assemble and form the transparent hydrogel [21].

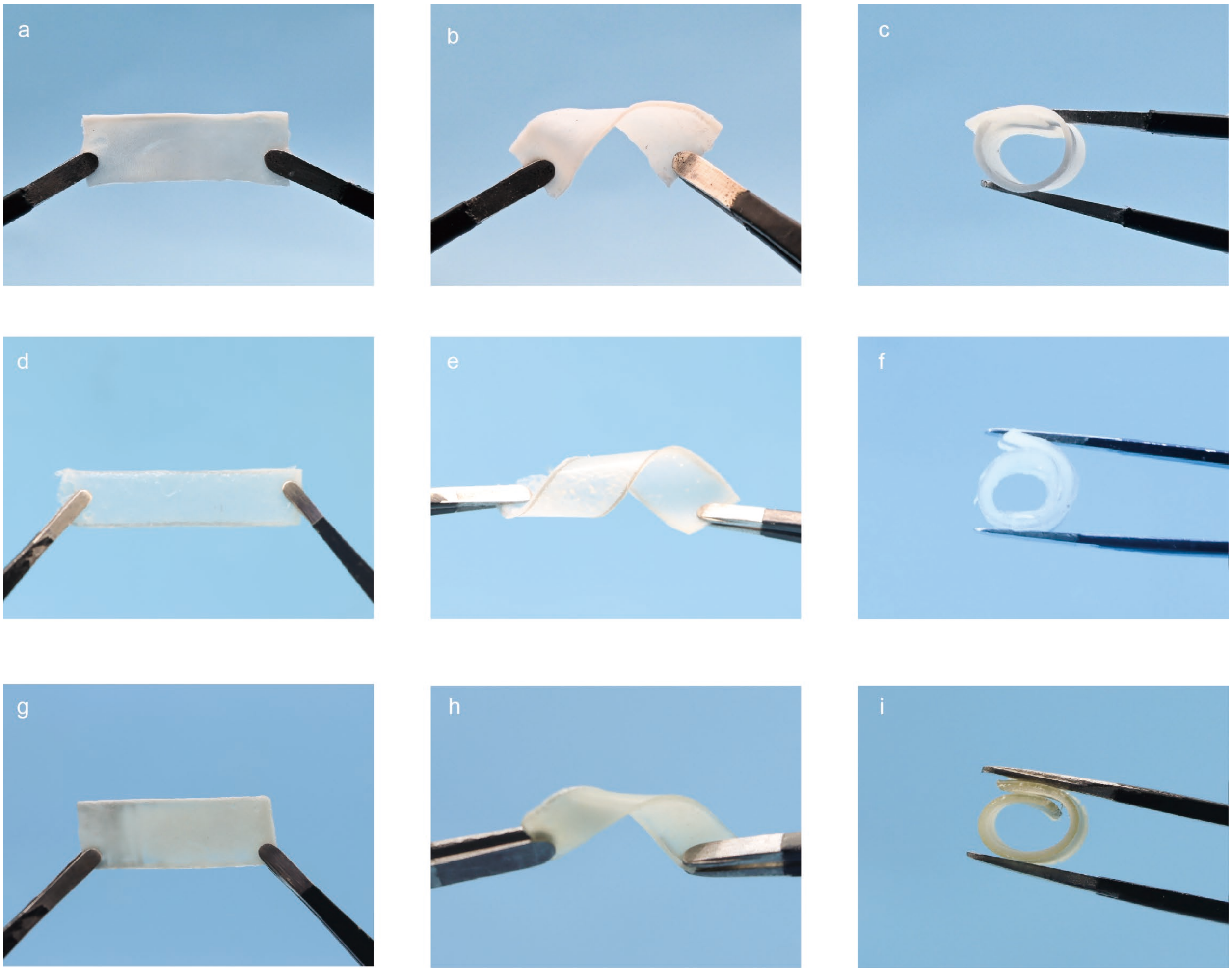

Figure S8. Optical photographs of (a) original, (b) twisted, and (c) rolled E-CMC-GE, (d) original, (e) twisted, and (f) rolled E-SA-GE, and (g) original, (h) twisted, and (i) rolled E-SF-GE.

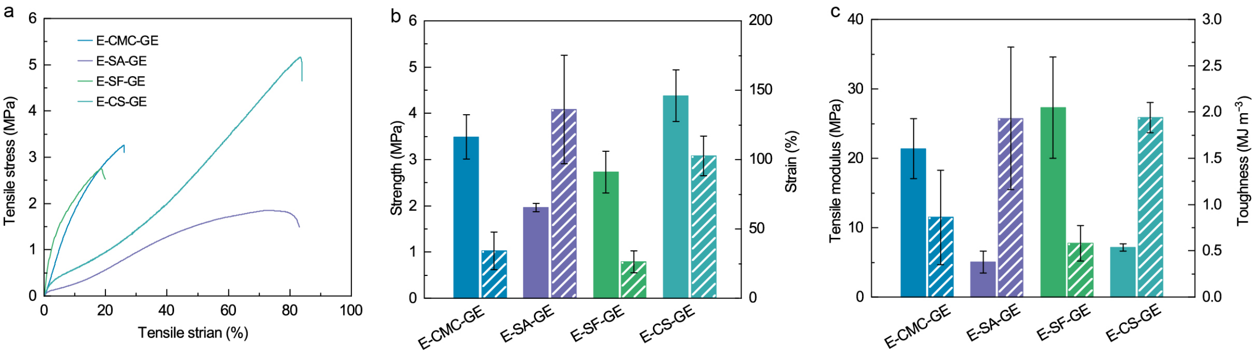

Figure S9. (a) Typical tensile stress−strain curves, (b) tensile strength and strain, and (c) tensile modulus and toughness of E-CMC-GE, E-SA-GE, E-SF-GE, and E-CS-GE.

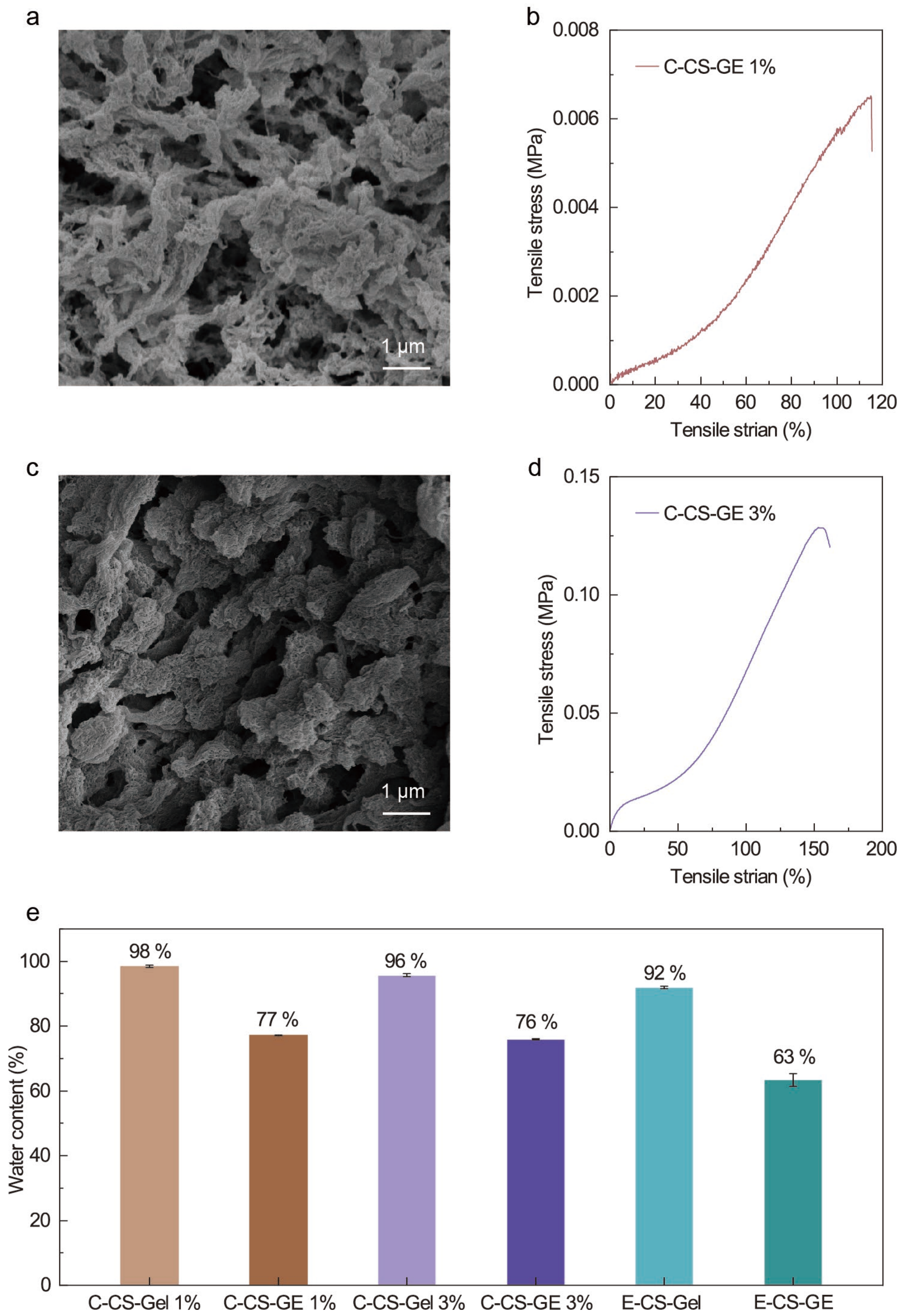

Figure S10. (a) An SEM image of the C-CS-GE 1 wt% morphology. (b) Tensile strength of the C-CS-GE 1 wt%. (c) An SEM image of the C-CS-GE 3 wt% morphology. (d) Tensile strength of the C-CS-GE 3 wt%. (e) Water content of CS hydrogels before and after immersion in 3 M $ZnSO_4$ solution.

To unravel the mechanisms behind the superior mechanical properties of the E-CS-GE, its compositions and microstructure were examined, in comparison to chitosan-based hydrogel electrolytes prepared via a solution casting method (The water solubility limit of CS is approximately 3 wt%).

As shown in Fig. S10a-d, the C-CS-GE samples prepared with CS solutions with concentrations of 1 wt% and 3 wt% both show a loose and irregular microstructure, which consequently leads to low tensile strengths of 6.5 and 128.4 kPa, respectively. The measured water contents of C-CS-Gel are 98% and 96%. After being soaked in 3 M $ZnSO_4$, the water content reduces to 77% (as depicted in Fig. S10e). The E-CS-GE in Fig. 1d and S9 exhibited a much denser structure with uniformly dispersed nanopores, thereby yielding a significantly increased tensile strength up to 4 MPa. Specifically, the water content of the E-CS-Gel is 92%, and it drops to 63% after soaking in 3 M $ZnSO_4$ (Fig. S10e). This is attributed to the fact that during the electrogelation process, the CS chains are tightly bound by hydrogen bonds under the influence of the electric field effect. As a result, the water content of E-CS-GE is lower than that in traditional casting hydrogels, resulting in better mechanical performance.

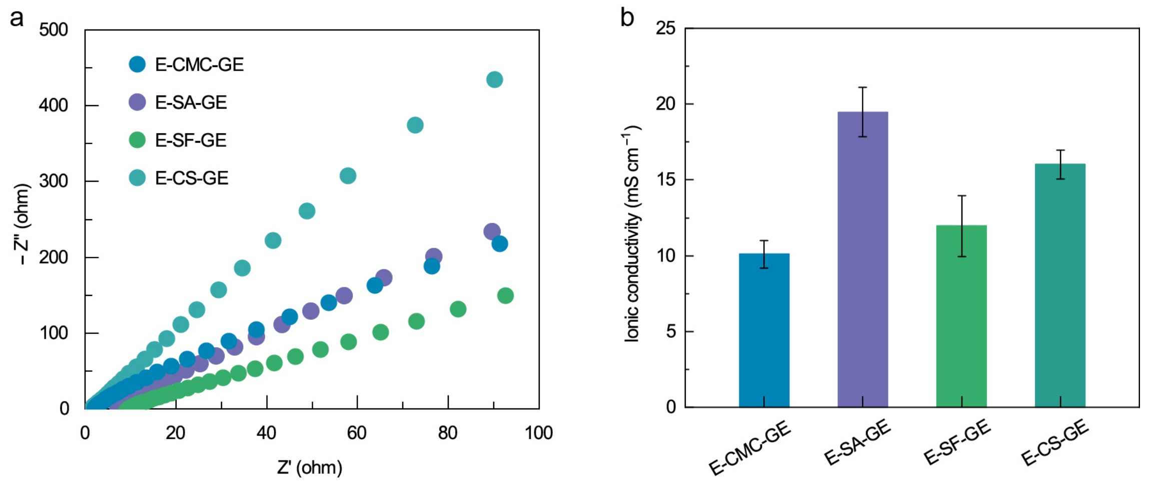

Figure S11. (a) Typical Nyquist plots and (b) ionic conductivities of E-CMC-GE, E-SA-GE, E-SF-GE, and E-CS-GE.

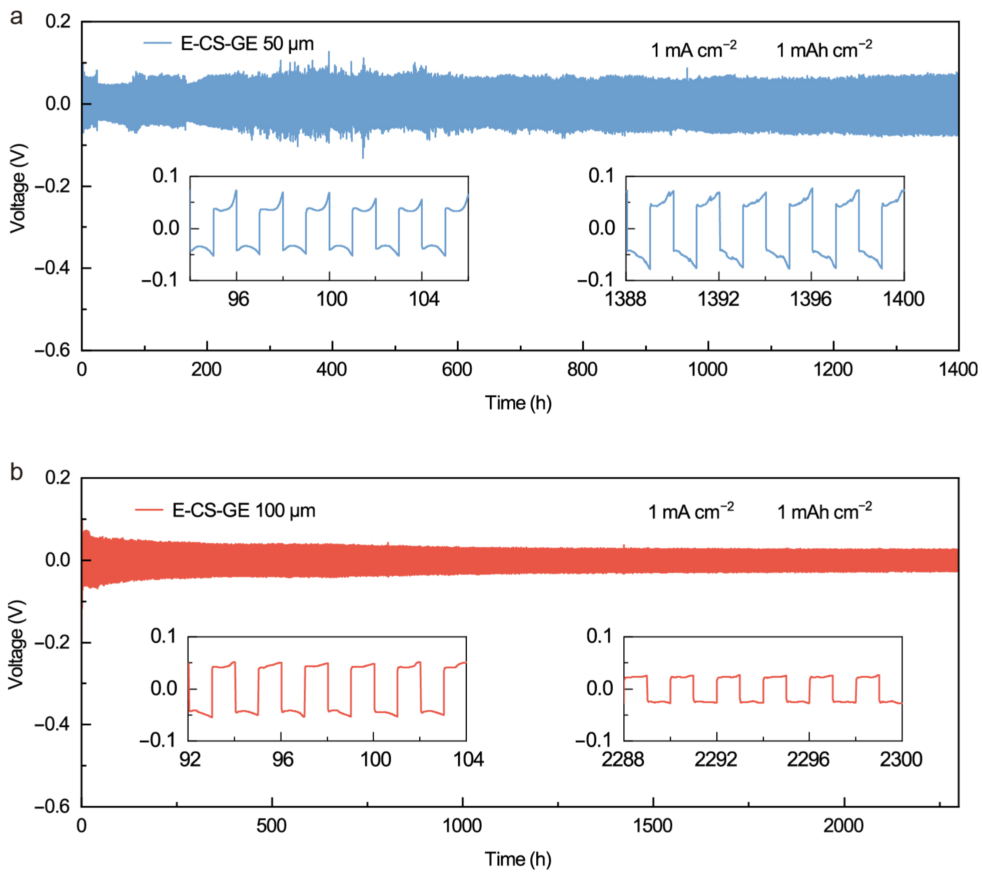

Figure S12. Galvanostatic Zn plating/stripping in Zn||Zn symmetric cells with E-CS-GE of thicknesses (a) 50 μm and (b) 100 μm.

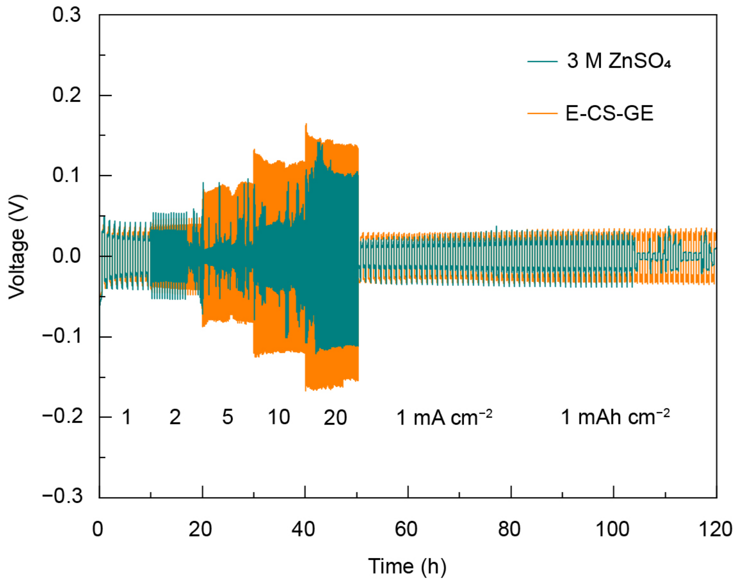

Figure S13. Rate capability of Zn||Zn symmetric cells at current densities of 1, 2, 5, 10, 20 mA cm^{-2} with a fixed deposition capacity of 1 mAh cm^{-2}.

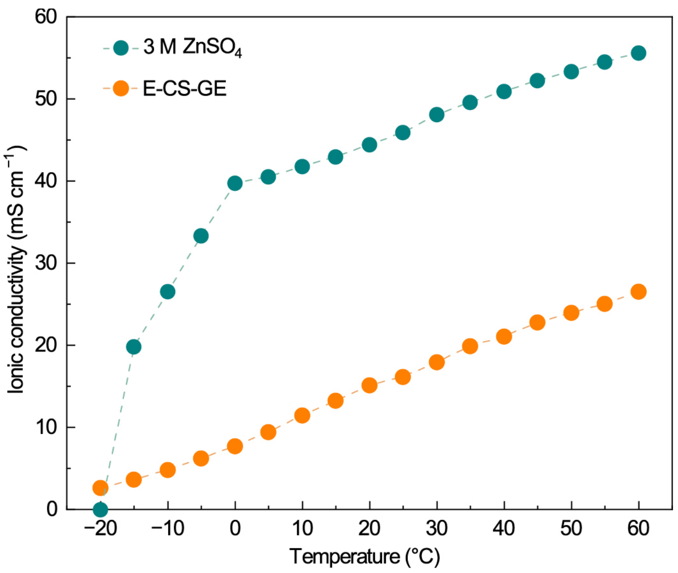

Figure S14. Ionic conductivity of 3 M $ZnSO_4$ and E-CS-GE in the temperature range between −20 and 60ºC.

As shown in Fig. S14, 3 M $ZnSO_4$ exhibits higher ionic conductivity than the E-CS-GE from −15 and 60ºC, attributed to unrestricted ion diffusion in free water. The CS polymer network of E-CS-GE acts as an obstacle course, increasing resistance and potentially trapping some ions, reducing overall mobility. However, its bound water-dominated structure maintains linear decrease from 60 to −20ºC, retaining 2.7 mS cm^{-1} at −20ºC, which surpasses that of the frozen liquid counterpart. These results reveal that the E-CS-GE possesses a superior freeze resistance, enabled by polymer-bound water suppressing ice nucleation.

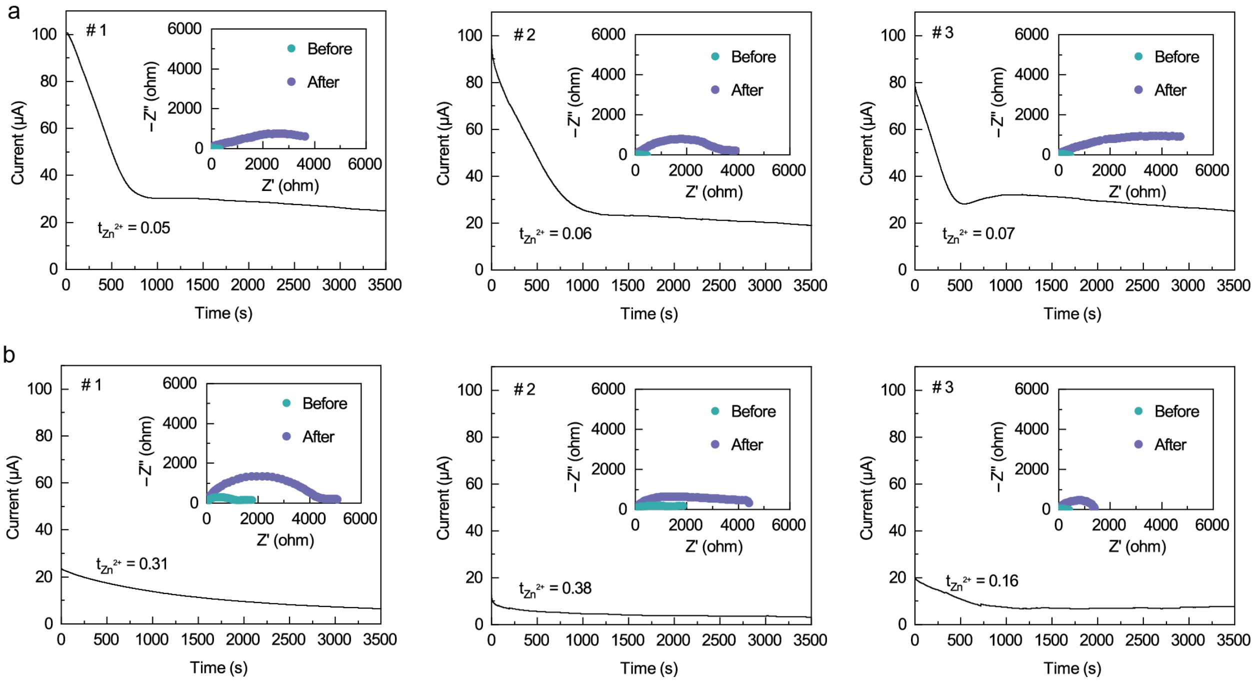

Figure S15. Measurements of Zn^{2+} transference numbers via the Bruce-Vincent method. Polarization curves of Zn||Zn cells with (a) 3 M $ZnSO_4$ (GF as separator) and (b) the E-CS-GE at a voltage bias of 10 mV. The insets are the EIS curves before and after polarization.

In our tests (Fig. S15), the I-t curves show good repeatability, while the evolution of interfacial resistance is not so regular, with all operations strictly following the well-documented testing protocol. The t_+ value is 0.06 ± 0.01 for 3 M $ZnSO_4$ (0.05, 0.06, and 0.07 in 3 trials). The I-t curve for the E-CS-GE shows a steady decline, which usually indicates a higher Zn^{2+} transference number. Eventually, the t_+ was calculated to be 0.28 ± 0.11 (0.31, 0.38, and 0.16 in 3 trials). Even though we experimentally obtained the t_+ of 3 M $ZnSO_4$ and the E-CS-GE, we prefer that they can only provide a reference value instead of being considered as the true results. The higher Zn^{2+} transference number is attributed to the selective regulation of ion mobility by E-CS-GE, which selectively immobilizes anions via electrostatic binding while relatively facilitating Zn^{2+} transport.

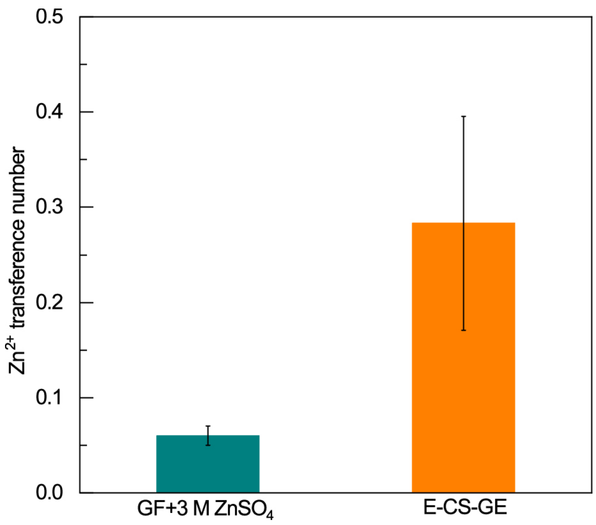

Figure S16. Zn^{2+} transference numbers for GF separator with 3 M $ZnSO_4$ and E-CS-GE.

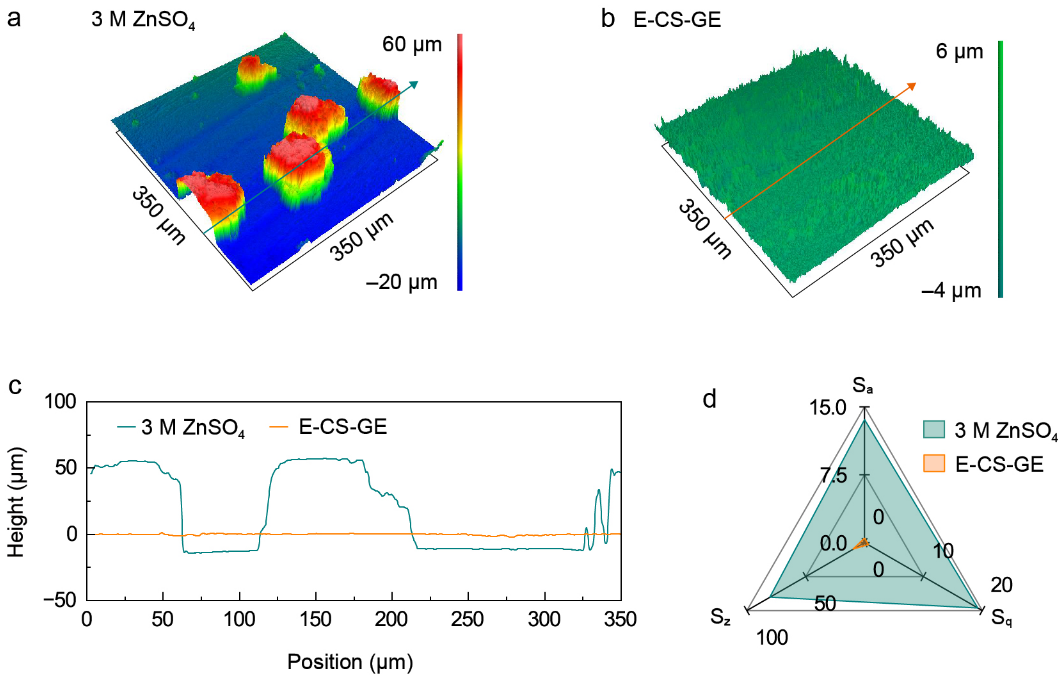

Figure S17. 3D height images of Zn deposition on Zn plates in (a) 3 M $ZnSO_4$ and (b) E-CS-GE. (c) The corresponding surface roughness curves and (d) surface parameters (S_a: surface arithmetic mean deviation, S_z: surface height of irregularities, and S_q: surface root mean square deviation).

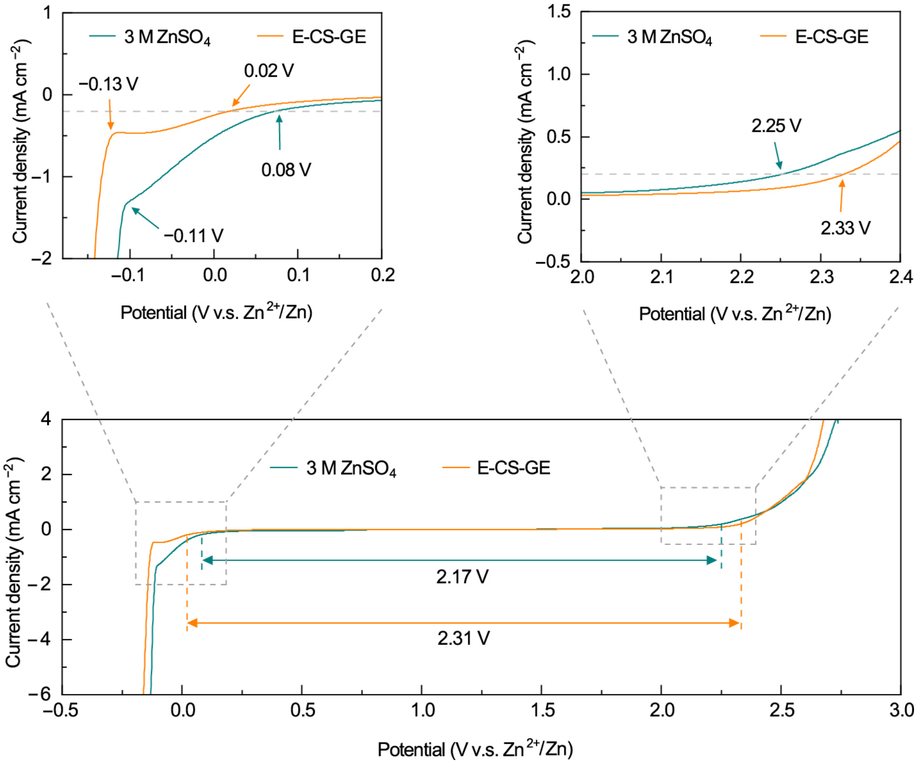

Figure S18. The electrochemical window of 3 M $ZnSO_4$ and the E-CS-GE, measured by LSV at a scan rate of 1 mV s^{-1}.

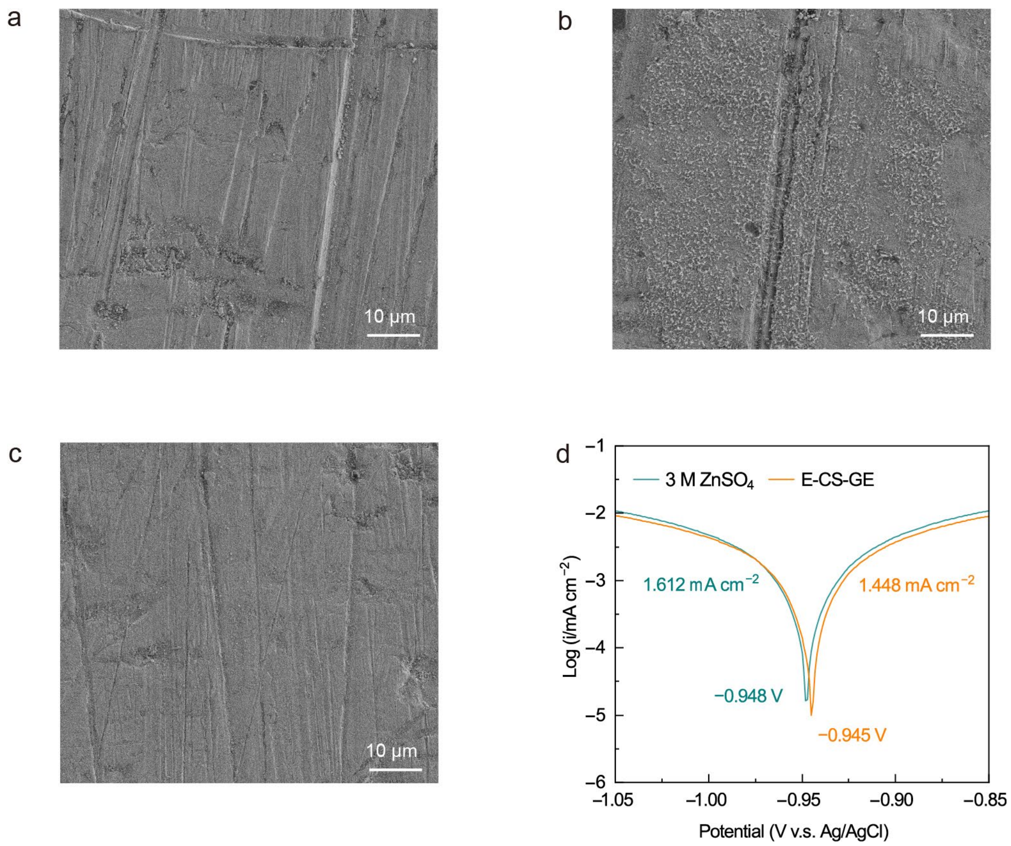

Figure S19. SEM images of (a) pristine Zn foil, (b) Zn foil after soaking in 3 M $ZnSO_4$ solution for 24 h, and (c) Zn foil coated with E-CS-GE after soaking in 3 M $ZnSO_4$ solution for 24 h. The E-CS-GE was peeled off before SEM observation. (d) Tafel curves of the bare Zn anodes and Zn anodes with E-CS-GE in 3 M $ZnSO_4$ tested using a three-electrode system.

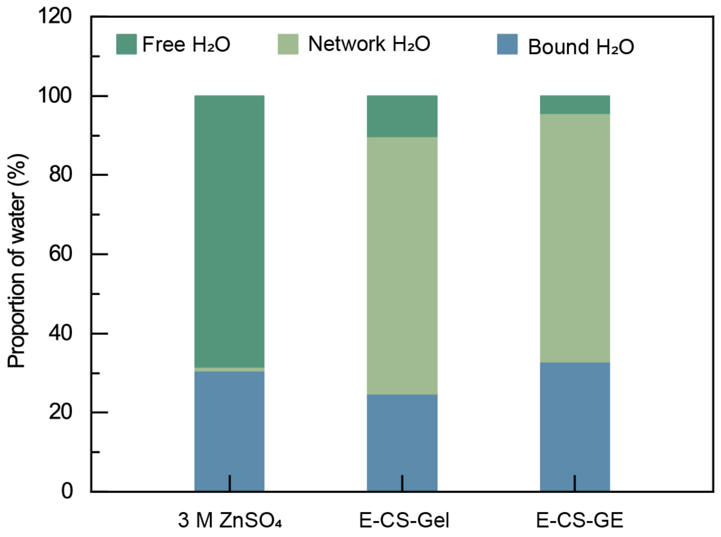

Figure S20. Proportion of water in 3 M $ZnSO_4$, the E-CS-Gel, and the E-CS-GE as determined by LF-NMR.

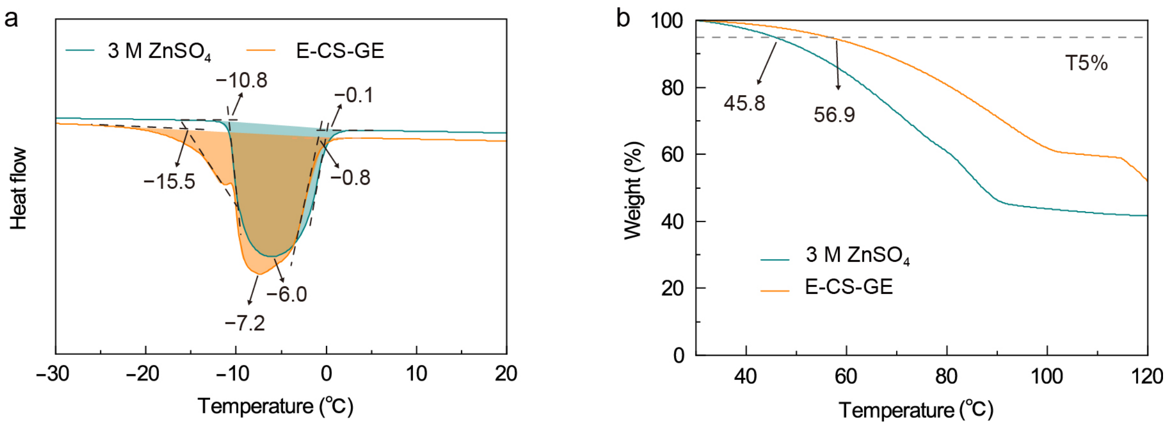

Figure S21. (a) DSC and (b) TGA curves of 3 M $ZnSO_4$ and E-CS-GE.

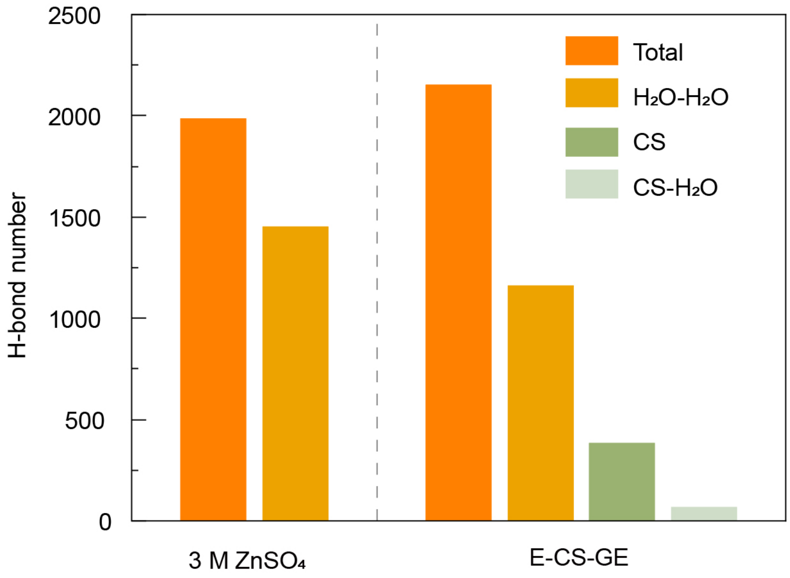

Figure S22. The average H-bond number between various parts in 3 M $ZnSO_4$ and the E-CS-GE.

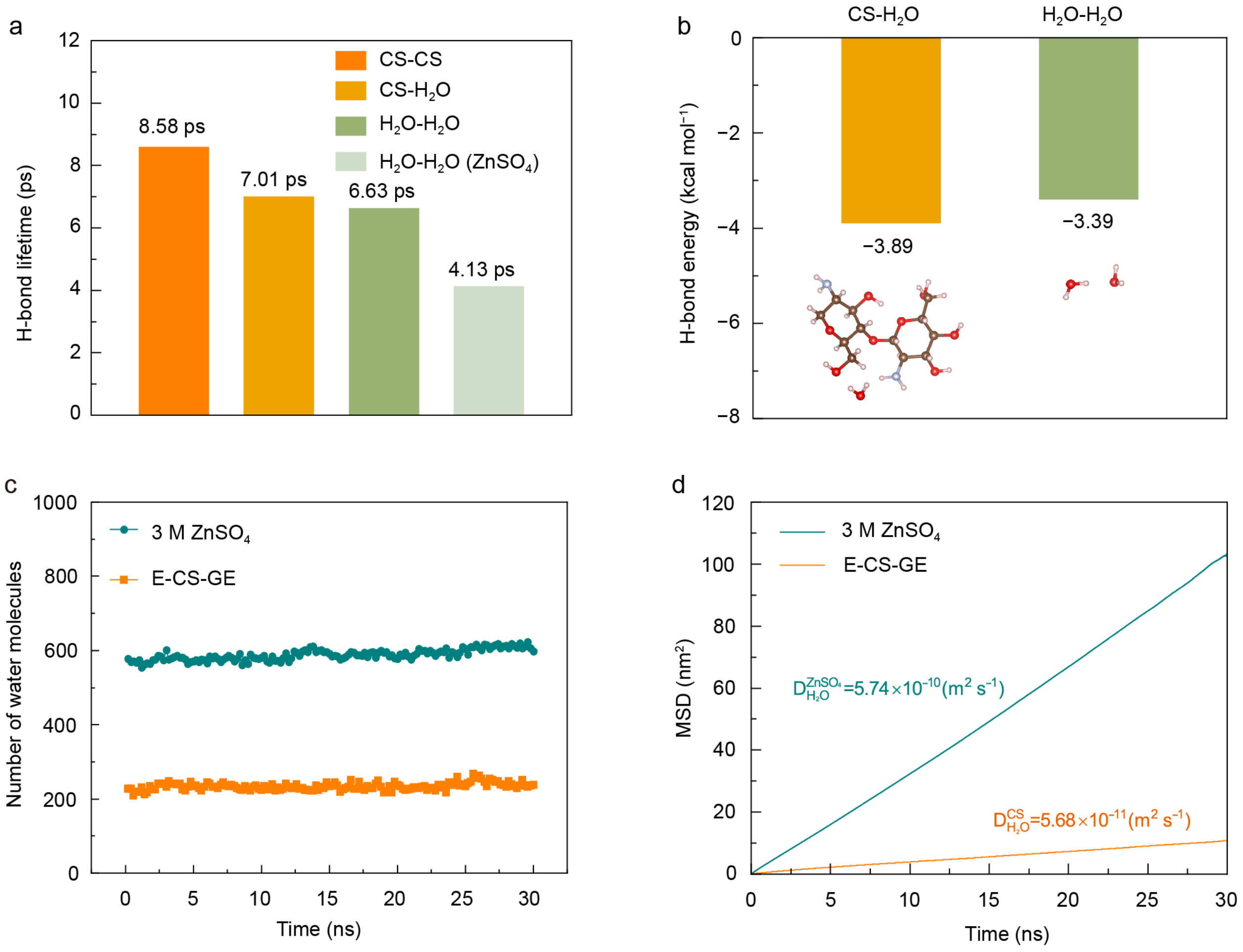

Figure S23. (a) H-bond lifetime between various components in 3 M $ZnSO_4$ and the E-CS-GE. (b) Binding energy between CS chain and H_2O molecule. (c) Number of free H_2O molecules and (d) diffusion coefficient of H_2O molecule in 3 M $ZnSO_4$ and the E-CS-GE.

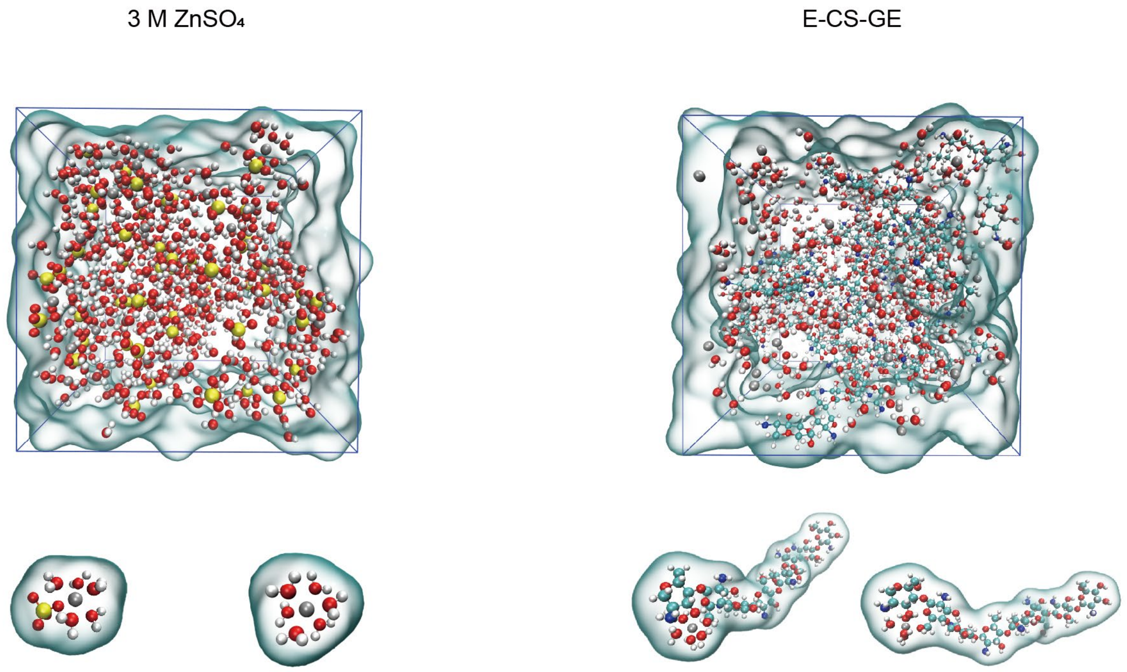

Figure S24. 3D snapshots of the MD simulations for 3 M $ZnSO_4$ and E-CS-GE, together with the corresponding schematics of Zn^{2+} solvent sheath.

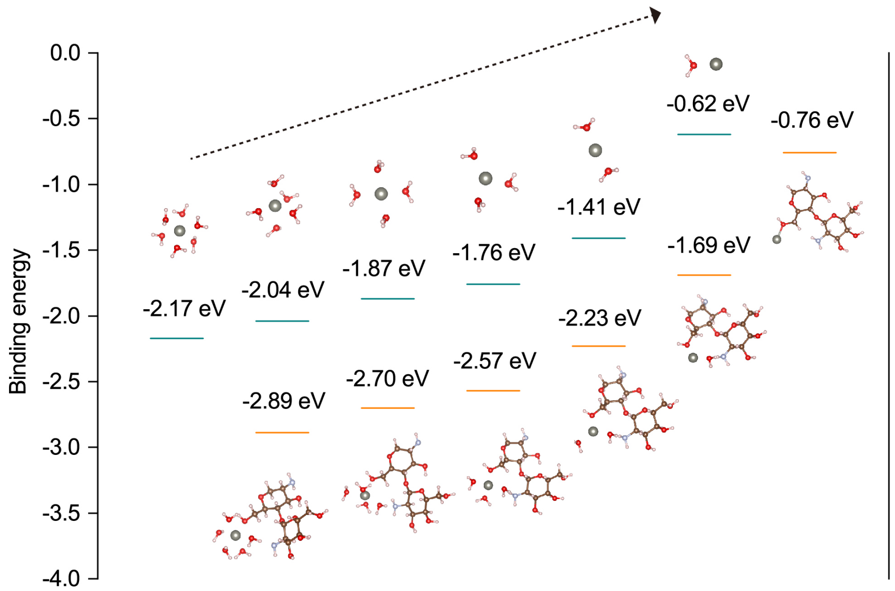

Figure S25. Desolvation energy of solvated Zn^{2+} in $ZnSO_4$ and E-CS-GE as calculated by DFT.

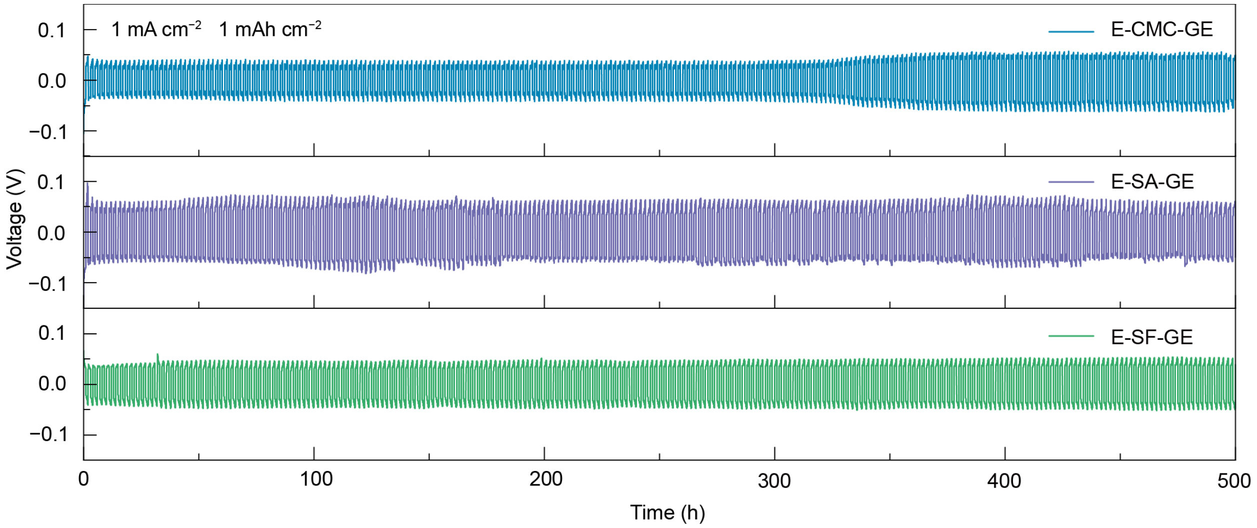

Figure S26. Electrochemical performance of the E-CMC-GE, E-SA-GE, and E-SF-GE electrolytes in Zn||Zn symmetric cells at a current density of 1 mA cm^{-2} with 1 mAh cm^{-2}.

The Zn||Zn symmetric cells were assembled to evaluate the cycling stability of E-GE. As presented in Fig. S26, the life spans of cells with the E-CMC-GE, E-SA-GE, and E-SF-GE are all over 500 h at the current density of 1 mA cm^{-2} and a capacity of 1 mAh cm^{-2}, significantly outperforming the liquid electrolyte (150 h). These results confirm the universal applicability of the *in situ* electrogelation strategy, which enables the design of multifunctional and high-performance hydrogel electrolytes from a variety of sustainable biomass resources for aqueous energy storage devices.

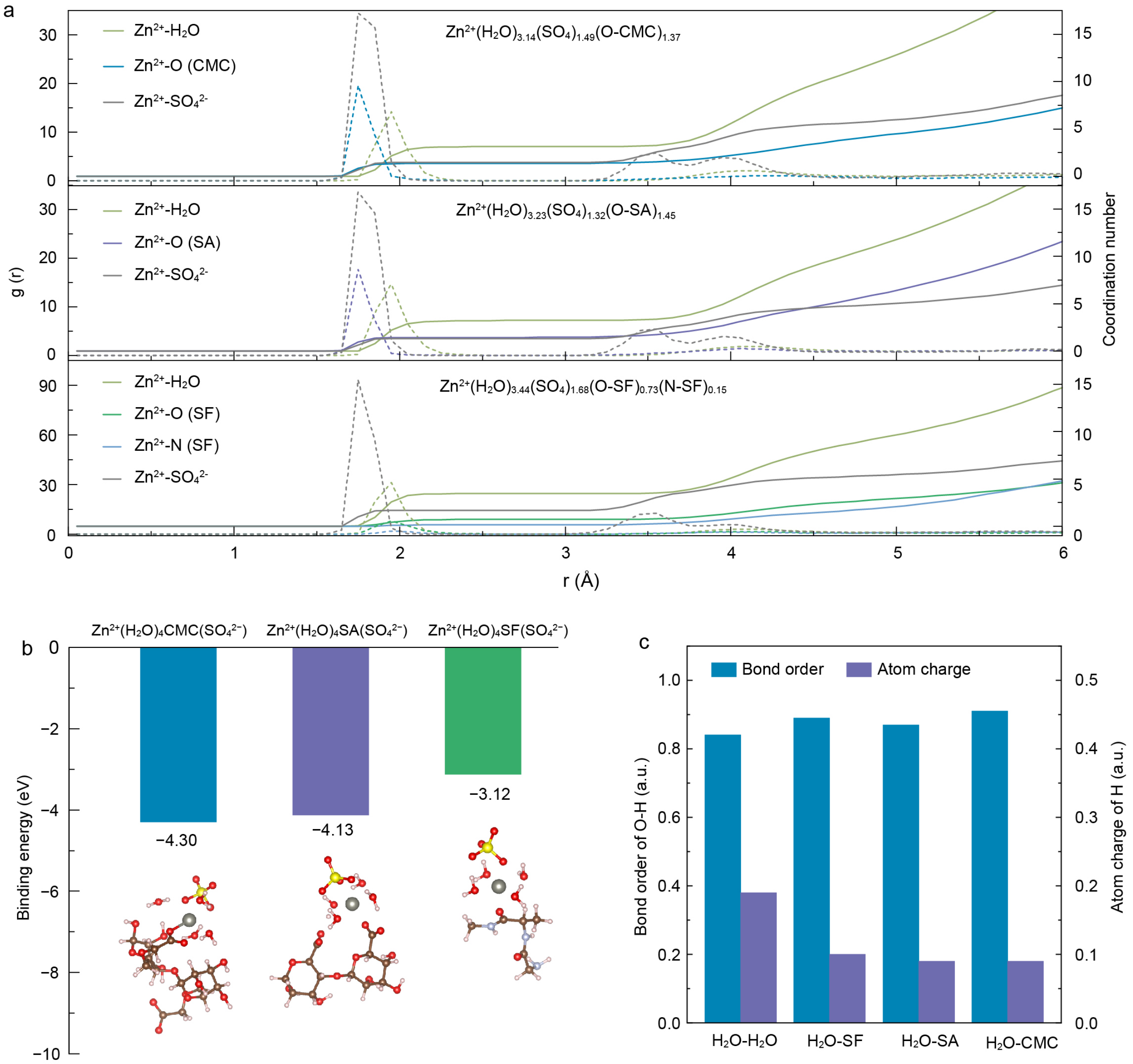

Figure S27. (a) Radial distribution function ($g(r)$) and coordination number (n(r)) curves of the E-CMC-GE, E-SA-GE, and E-SF-GE. (b) Calculated binding energies of Zn^{2+}-CMC, Zn^{2+}-SA, and Zn^{2+}-SF. (c) O–H bond orders and average atomic charges of H in H_2O-H_2O, H_2O-SF, H_2O-SA, and H_2O-CMC configurations.

To further unveil the solvation structure of Zn^{2+} in E-GE, MD simulations were performed. The RDF and CN of E-CMC-GE, E-SA-GE, and E-SF-GE (Fig. S27a) show that the corresponding solvation structures are $Zn^{2+}(H_2O)_{3.14}(SO_4^{2-})_{1.49}(CMC)_{1.37}$, $Zn^{2+}(H_2O)_{3.23}(SO_4^{2-})_{1.32}(SA)_{1.45}$, and $Zn^{2+}(H_2O)_{3.44}(SO_4^{2-})_{1.68}(SF)_{0.88}$, respectively. These results

demonstrate that three kinds of biomacromolecules are involved in the solvation structures of Zn^{2+}, respectively, regulating the transportation of Zn^{2+}. For mechanistic comparison, the common solvation structures of the Zn^{2+} in the E-GE and liquid electrolytes were simplified as $Zn^{2+}(H_2O)_4(SO_4^{2-})$(CMC), $Zn^{2+}(H_2O)_4(SO_4^{2-})$(SF), and $Zn^{2+}(H_2O)_4(SO_4^{2-})$(SA) (Fig. S27b). The calculated binding energies of corresponding solvation structures are −4.30, −4.13, and −3.12 eV, respectively, verifying the enhanced desolvation capacity in E-GE due to biopolymer coordination. In addition, the stable binding structure of the H_2O molecule and polymer chain was further established to illustrate the suppressed activity of H_2O by analyzing the atomic charge of hydrogen and the O–H bond order of the structure. As shown in Fig. S27c, the O–H bond orders of H_2O-SF (0.89), H_2O-SA (0.87), and H_2O-CMC (0.91) are higher than that of H_2O-H_2O (0.84), and the average atomic charges of the H atoms in H_2O-SF (0.10), H_2O-SA (0.09), and H_2O-CMC (0.09) are lower than that in H_2O-H_2O (0.19). These findings indicate biopolymers stabilize water molecules through hydrogen bonding, suppressing water activity and thereby inhibiting HER in E-GE [7].

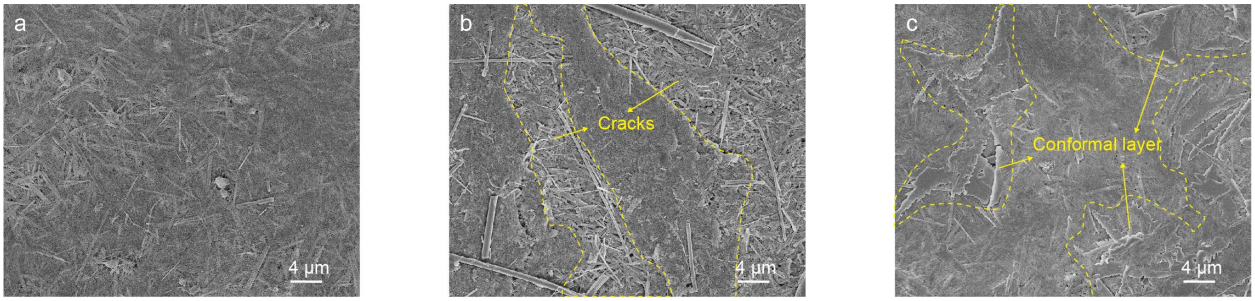

Figure S28. SEM images of ZVO electrodes: (a) pristine, (b) after cycling in 3 M $ZnSO_4$, and (c) after cycling in the E-CS-GE.

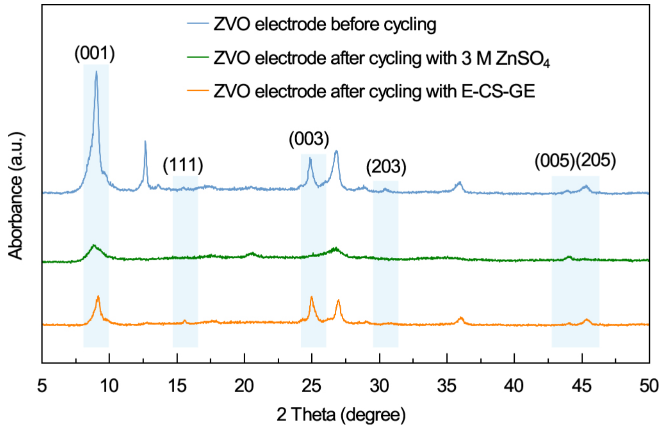

Figure S29. XRD patterns of pristine and cycled ZVO electrodes.

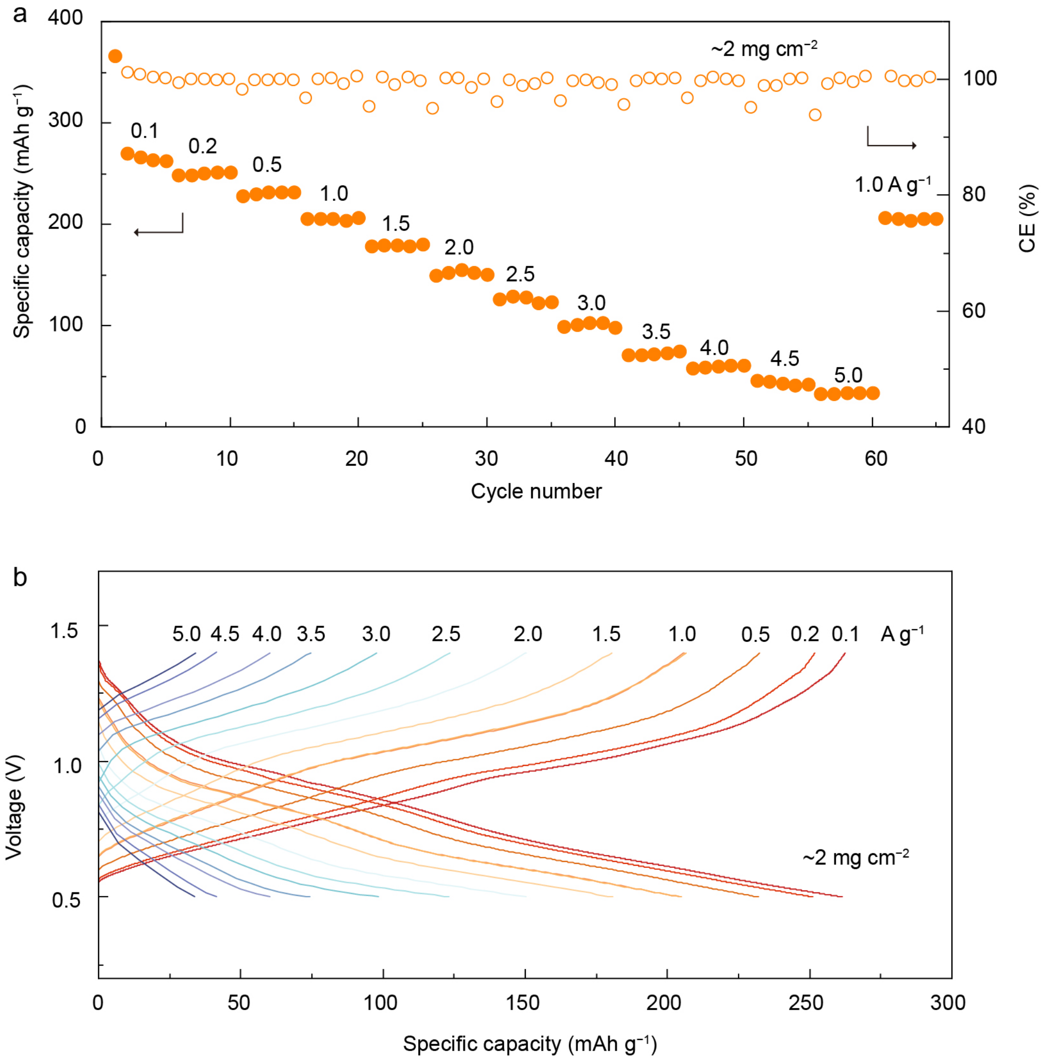

Figure S30. (a) Rate capability and (b) galvanostatic charge and discharge potentials of the Zn||ZVO cell with E-CS-GE at different current densities.

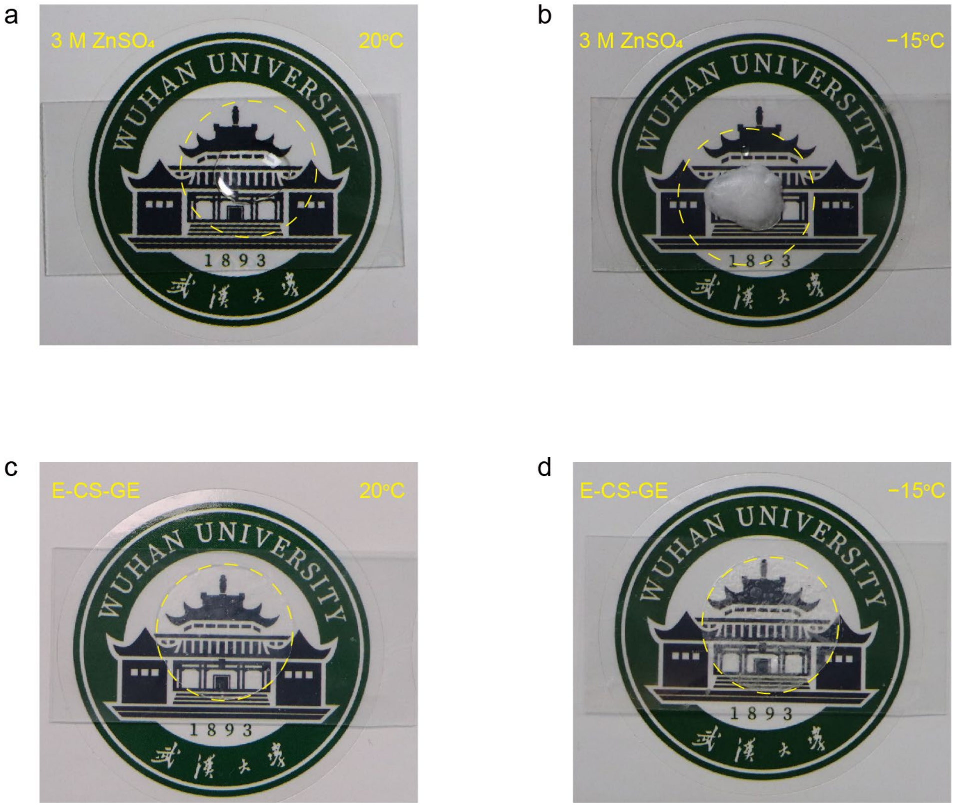

Figure S31. Optical photos of 3 M $ZnSO_4$ liquid electrolyte at (a) 20°C and (b) −15°C. Optical photos of the E-CS-GE at (c) 20°C and (d) −15°C.

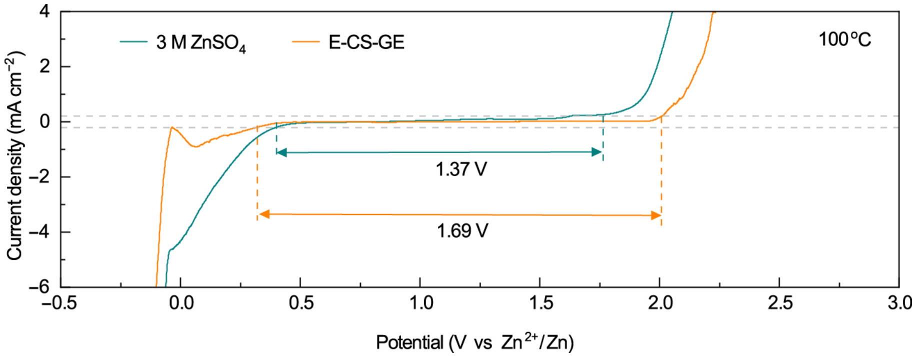

Figure S32. Electrochemical stability window of 3 M $ZnSO_4$ and the E-CS-GE measured by LSV at a scan rate of 1 mV s^{-1} at 100°C.

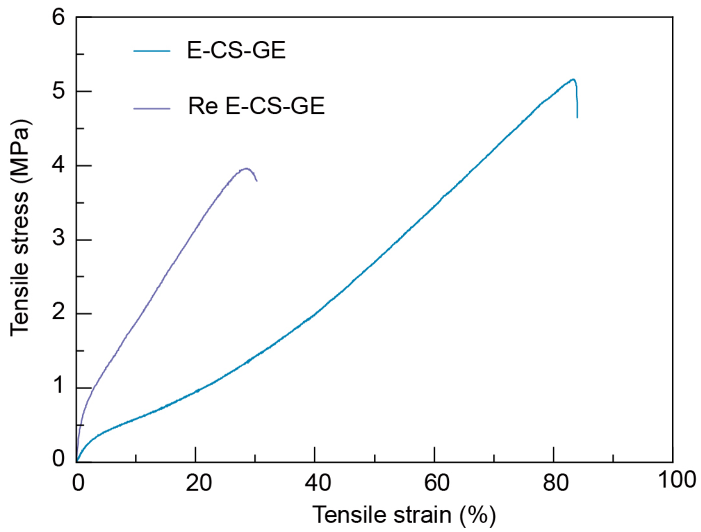

Figure S33. Stress-strain curves of the E-CS-GE before and after regeneration.

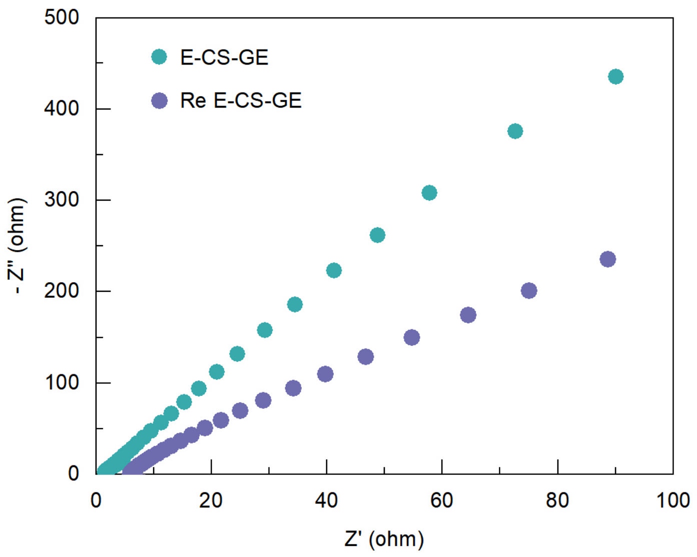

Figure S34. Nyquist plots of the E-CS-GE and Re E-CS-GE.

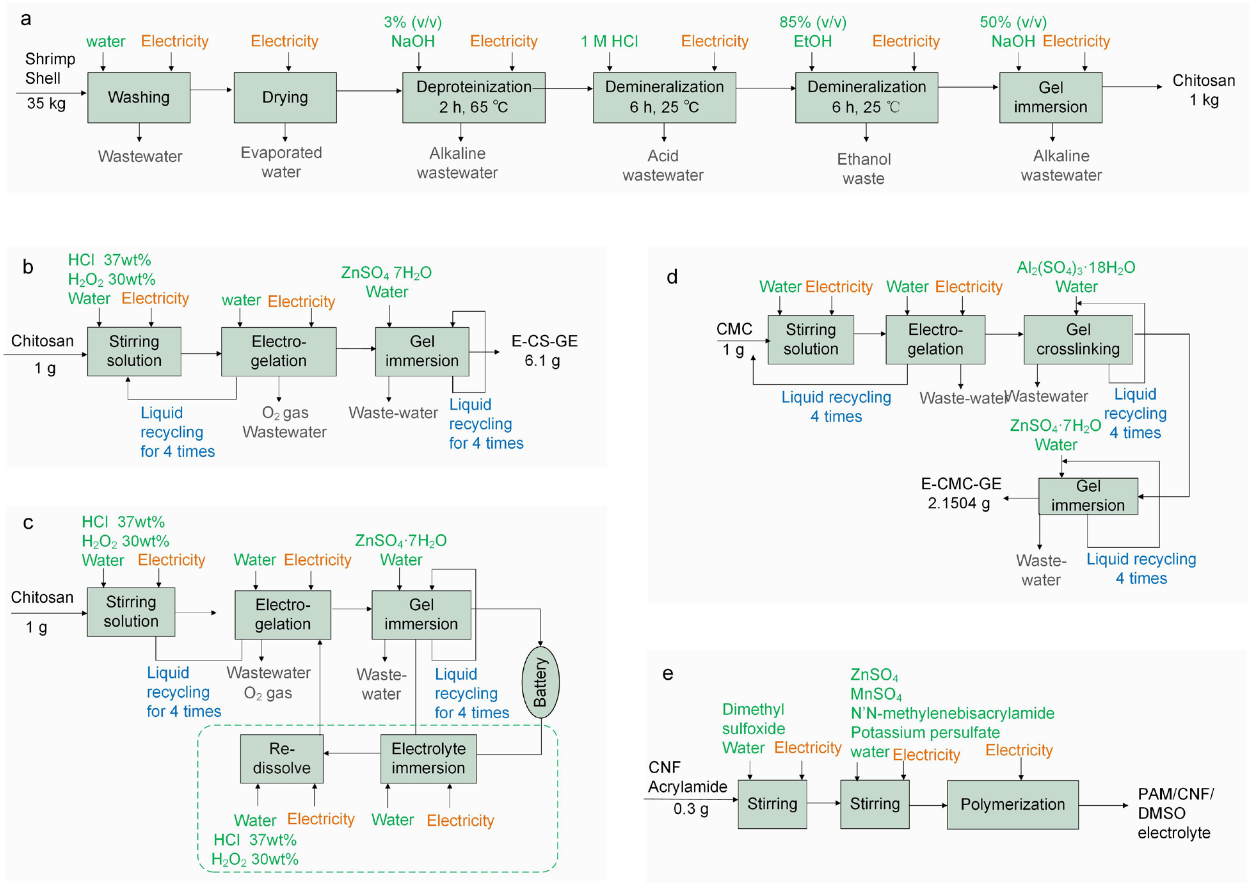

Figure S35. Flowchart for the fabrication of (a) "*CS*", (b) "*E-CS-GE*", (c) the recycling of "*E-CS-GE*", (d) "*E-CMC-GE*", (e) "*PAM/CNF/DMSO electrolyte*". Material inputs are highlighted in pale green, electricity need is highlighted in orange, the recycled materials are highlighted in blue, wastes are highlighted in grey, processes are highlighted with a green box, and the recycling process of "*E-CS-GE*" is highlighted within a green-lined box.

Table S1. Mechanical strength, ionic conductivity, CE, thermal stability, recyclability, and cycling life of the E-CS-GE and reported hydrogel electrolytes of Zn-Alg and PAN/CNF/DMSO.

Electrolyte	Tensile strength (MPa)	Ionic conductivity (mS cm^{-1})	CE (%)	Cycling life	Thermal stability	Recycl ability	Refs.
Zn-Alg	1.16^{E}	25	99.65	500	100^{E}	8.0	7
PAM/CNF/ DMSO	0.0554	13.3	99.1	1450	45	5.0	8
E-CS-GE	4.4	16.0	99.83	4500	125	10.0	This work

^{E}: The value is evaluated in our lab.

Table S2. Comparison of ionic conductivity and mechanical ability of the E-CS-GE, E-CMC-GE, E-SA-GE, and E-SF-GE with literature. PVHF: poly(vinylidene fluoride-cohexafluoropropylene), CPZ-H: CS- and polyaspartic acid-containing $ZnSO_4$ hydrogel, ChNF: chitin nanofiber, and LPH: lignin-containing cellulose nanofiber-polyacrylamide hydrogel.

Electrolyte	Ionic conductivity ($mS\ cm^{-1}$)	Tensile strength (MPa)	Ref.
PVHF gel	1.14	2.10	10
Dough gel	2.5	0.0651	11
PAM gel	3.93	0.446	12
CPZ-H gel	5.51	0.0532	13
Cellulose gel	10.6	0.220	14
PAM/CNF/DMSO gel	13.3	0.0554	8
PAM–ChNF gel	15.2	0.11462	15
LPH gel	21.57	0.350	16
E-CS-GE	16.0	4.4	This work
E-CMC-GE	10.1	3.5	This work
E-SA-GE	19.5	2.0	This work
E-SF-GE	12.0	2.7	This work

Table S3. Comparison of overpotentials of E-CS-GE with previously reported hydrogel electrolytes in AZMBs (non-exhaustive survey). ZIG: zwitterion hydrogel electrolyte, HA: hyaluronic acid, and PCZ-gel: an amphoteric hydrogel electrolyte consisting of PAM, carboxylethyl quaternized cellulose, and $ZnSO_4$ salt.

Hydrogel electrolytes	Ionic conductivity ($mS\ cm^{-1}$)	Current density ($mA\ cm^{-2}$)	Overpotential (mV)	Refs
ZIG-20 wt%	2.6	1	~120	17
HA gel	47.7	1	50	18
PAM/LiCl	N/A	1	80	19
PAM gel	3.93	1	~65	12
PCZ-gel	27.5	1	~41	20
		20	70	
Cellulose gel	10.6	1	~77	14
		10	~81	
E-CS-GE	16.0	1	36.3	This work
		4.7	57.4	
		20	111.8	

Table S4. Comparison of cycling life for Zn||Zn symmetric cells between this work and literature. PVDF: polyvinylidene difluoride, NTP: sodium tricyanomethanide and polyacrylonitrile, AG: agarose, TSAE: temperature self-adaptive electrolyte, PVA: polyvinyl alcohol, SR: SO_4^{2-} receptor, ZP@Zn: a SEI layer on anodes, PA: polyamide, and $Zn(OTf)_2$: zinc trifluoromethanesulfonate.

	Electrolyte	Current density (mA cm^{-2})	Capacity density (mAh cm^{-2})	Cycling life (h)	DOD (%)	Ref.
PVDF-Sn@Zn coating layer	2 M $ZnSO_4$	1<br>10	1<br>10	1200<br>200	–<br>–	21
$La(NO_3)_3$ additive	2 M $ZnSO_4$	1<br>10	1<br>5.93	1200<br>160	–<br>80	22
NTP layer	2 M $ZnSO_4$	1<br>20	1<br>5	1500<br>125	–<br>–	23
Bi/Bi_2O_3 layer	2 M $ZnSO_4$	1<br>10	1<br>10	3120<br>300	2.6<br>60	24
AG/CMC gel electrolyte	Gel based on 2 M $ZnSO_4$	1	1	4100	–	25
HA gel electrolyte	Gel based on 2 M $ZnSO_4$	1<br>6.5	1<br>6.5	5500<br>250	–<br>80	18
TSAE electrolyte	2 M $Zn(OTf)_2$ in H_2O/tetraglyme	1	1	16800	–	26
CPZ-H gel electrolyte	Gel based on 2 M $ZnSO_4$	25<br>32.8	25<br>32.8	900<br>120	–<br>80	13
PVA@SR-$ZnMoO_4$	Gel based on 2 M $ZnSO_4$	10	10	275	–	27
ZP@Zn layer	2 M $ZnSO_4$	20<br>26.55	5<br>21.74	470<br>60	–<br>80	29
BC gel electrolyte	Gel based on 2 M $ZnSO_4$	6.5	6.5	100	85	30
Zn with 101 texture	2 M $ZnSO_4$	15	10	330	–	28
PA/$Zn(OTf)_2$ layer	2 M $ZnSO_4$	10	10	150	85	31
PCZ-gel electrolyte	Gel based on 2 M $ZnSO_4$	9.11	9.11	440	80	20

E-CS-GE	Gel based on 3 M $ZnSO_4$	1	1	4520	−	This work
		20	5	450	−	
		4.7	4.7	300	80	

Table S5. Comparison of cumulative capacity and CE for Zn||Cu asymmetric cells using the E-CS-GE with literature.

	Electrolyte	Current density (mA cm^{-2})	Capacity density (mAh cm^{-2})	Cumulative capacity (Ah cm^{-2})	CE (%)	Ref.
HA gel electrolyte	Gel based on 2 M $ZnSO_4$	1	1	2	99.7	18
CPZ-H gel electrolyte	Gel based on 2 M $ZnSO_4$	10	5	4.25	99.6	13
PVDF-Sn@Zn coating layer	2 M $ZnSO_4$	5	1	0.5	99.0	21
NTP layer	2 M $ZnSO_4$	1	1	0.3	99.2	23
Zinc with 101	2 M $ZnSO_4$	15	10	1.65	99.3	28
ZP@Zn layer	2 M $ZnSO_4$	1	1	0.35	98.3	29
BC gel electrolyte	Gel based on 2 M $ZnSO_4$	2	1	1	99.7	30
PCZ-gel electrolytes	Gel based on 2 M $ZnSO_4$	2	2	3	99.4	20
E-CS-GE	Gel based on 3 M $ZnSO_4$	1	1	2.8	99.8	This work

Table S6. Parameters and compositions for molecular dynamics (MD) simulations of E-GE and liquid electrolytes.

	3 M $ZnSO_4$
Number of Zn^{2+} per box	60
Number of SO_4^{2-} per box	60
Number of H_2O per box	1060
Number of polymer chains per box	0
Total number of atoms	3540
Simulation box size (Å^3)	40.3×40.3×40.3
Temperature (K)	293.15

	CS
Number of Zn^{2+} per box	60
Number of SO_4^{2-} per box	60
Number of H_2O per box	1000
Number of polymer chains per box	10
Total number of atoms	5140
Simulation box size (Å^3)	51.3×51.3×51.3
Temperature (K)	293.15

	CMC
Number of Zn^{2+} per box	60
Number of SO_4^{2-} per box	60
Number of H_2O per box	1000
Number of polymer chains per box	9

Total number of atoms	5250
Simulation box size (Å^3)	52.3×52.3×52.3
Temperature (K)	293.15

	SF
Number of Zn^{2+} per box	60
Number of SO_4^{2-} per box	60
Number of H_2O per box	1000
Number of polymer chains per box	20
Total number of atoms	9920
Simulation box size (Å^3)	52.3×52.3×52.3
Temperature (K)	293.15

	SA
Number of Zn^{2+} per box	60
Number of SO_4^{2-} per box	60
Number of H_2O per box	1000
Number of polymer chains per box	14
Total number of atoms	5516
Simulation box size (Å^3)	50.3×50.3×50.3
Temperature (K)	293.15

Table S7. Comparison of the temperature adaptability of the full cells using the E-CS-GE with the previously reported gel electrolytes. HSAH: (R)-12-hydroxystearic acid hydrazide, PMAEDS: poly[2-(methacryloyloxy)ethyl]diethy-(3-sulopropyl), DES: deep eutectic solvents, ChCl: choline chloride, Gly: glycerol, PANI: Polyaniline, EG: ethylene glycol, SBMA: 2-(methacryloyloxy)ethyl] dimethyl-(3-sulfopropyl), PAMPS: poly(2-acrylamide-2-methylpropane sulfonic acid), KAc: potassium acetate, AN: acetonitrile, Agr: agarose, PEG: polyethylene glycol, and GG: guar gum.

Electrolytes	Liquid solution	Anti-freezing components	Ionic conductivity (mS cm^{-1})	Anode|| cathode	Working Temperature (°C)	Ref.
HSAH/PMAEDS/Zn-DES	$ZnCl_2$	ChCl, urea, EG	1.11 (−60°C)<br>10.36 (80°C)	Zn||MnO_2	80 (−20, 60)	36
PAM/Gly	$Zn(CF_3SO_3)_2$	Gly	0.0965 (−40°C)	Zn||PANI	60 (−40, 20)	37
PAM/EG	$Zn(CF_3SO_3)_2$	EG	17.6 (−20°C)<br>24.11 (RT)	Zn||V_2O_5	90 (−70, 20)	38
PAM/DMSO	$Zn(CF_3SO_3)_2$	DMSO	~1 (−40°C)<br>~69 (60°C)	Zn||$Zn_3V_2O_8$	100 (−40, 60)	39
CNF/PAM/SBMA	$Zn(CF_3SO_3)_2$	$Zn(CF_3SO_3)_2$	23.6 (−)	Zn||NVO	65 (−40, 25)	40
PAM/PAMPS	$Zn(ClO_4)_2$	$Zn(ClO_4)_2$	14.2 (−35°C)<br>59.9 (25°C)	Zn||PANI	60 (−35, 25)	41
PVA/DMSO/KAc	$Zn(Ac)_2$	KAc	0.01 (−80°C)<br>49.8 (25°C)	Zn||PANI	55 (−30, 25)	42
PAM/CNF/DMSO	$ZnSO_4$	DMSO	2.82 (−20°C)<br>13.3 (25°C)	Zn||MnO_2	45 (−20, 25)	8
PAM/Gly/AN	$ZnSO_4$	Gly	8.43 (−20°C)<br>13.94 (25°C)	Zn||V_2O_5	80 (−20, 60)	43
PAM/LiCl	$ZnSO_4$	LiCl, $ZnSO_4$	–	Zn||$LiFePO_4$	45 (−20, 25)	19
Agr/PEG	$ZnSO_4$	PEG	51.6 (RT)	Zn||NVO	70 (30, 100)	44
SA/GG/EG	$ZnSO_4$ + $MnSO_4$	EG	6.19 (−20°C)<br>16.81 (25°C)	Zn||MnO_2	45 (−20, 25)	45
E-CS-GE	$ZnSO_4$	CS, $ZnSO_4$	2.7 (−20°C)<br>26.5 (60°C)	Zn||ZVO	125 (−15, 110)	This work

Table S8. Comparison of the cycling performance of the full cells using the E-CS-GE with the previously reported gel electrolytes. FCOF: fluorinated covalent organic frameworks, COP: Covalent organic polymer, PMCNA: copolymerizing 2-methacryloyloxyethyl phosphorylcholine and N-acryloyl glycinamide, $Zn(DBS)_2$: zinc dodecylbenzenesulfonate, N,S-CDs: nitrogen and sulfurcodoped carbon quantum dots, Zn@Cd: Cd-coated Zn, HMPA: hexamethylphosphoramide, and $g\text{-}C_3N_4$: graphitic C_3N_4.

	Electrodes	N/P	Cumulative capacity (mAh cm^{-2})	Areal capacity (mAh cm^{-2})	Ref.
FCOF protective layer	Zn||MnO_2	10	578	1.36	46
	Zn||MnO_2	5	392	1.36	
COP protective layer	Zn||MnO_2	8	251	0.59	47
	Zn||MnO_2	1.2	53	0.59	
Maltose electrolyte additive	Zn||KVO	3	640	0.8	48
	Zn||KVO	3	174	1.46	
CNF/MXene protective layer	Zn||VO_2	2.8	237	2.1	49
PMCNA polymer additive	Zn||PANI	2.6	444	1.7	50
ZnF_2/Ag protective layer	Zn||PANI	2.3	361	5.0	51
$Zn(DBS)_2$-based eutectic electrolyte	Zn||NVP	2	27	0.31	52
N,S-CDs electrolyte additive	Zn||NVO	1.83	478	2.27	53
	Zn||NVO	1.05	181	3.38	
	Zn||NVO	1.46	328	4.99	
Zn@Cd anode	Zn||MnO_2	1.7	279	3.50	54
HMPA additive	Zn||V_6O_{13}	1.07	416	1.97	55

g-C_3N_4 colloidal electrolyte	Zn‖ZnVO	1.85	2520	6.30	56
E-CS-GE	Zn‖ZVO	1.1	483	5.45	This work

Table S9. Parameters of the pouch cell components.

Pouch cell components	Pieces	Length (mm)	Width (mm)	Weights (g)
Negative electrode (20 μm Zn foil) with E-CS-GE	2	106	96	16.15
Positive electrode (Ti mesh + ZVO)	3	102	92	13.52
Other encapsulation (aluminum plastic film, tab, etc.)	-	-	-	4.58

Table S10. Material and energy inventory for "*E-CS-GE*". The output from the previous step is highlighted in blue, and wastes are highlighted in light orange.

Comment	Item	Amount (g)	Source/provider
**Step 1: CS solution (100 g solution)**			
Raw materials	CS	0.5328 [a]	*Own process*
To dissolve CS	HCl solution	0.1011 [b]	market for hydrochloric acid, without water, in 30% solution state | hydrochloric acid, without water, in 30% solution state | Cutoff, U - RoW
0.2 g at 30% to avoid H_2 bubbles	H_2O_2 solution	0.1200	market for hydrogen peroxide, without water, in 50% solution state | hydrogen peroxide, without water, in 50% solution state | Cutoff, U - RoW
To dissolve CS	DI water	19.7180 [c]	market for water, deionised | water, deionised | Cutoff, U - RoW
Stirring electricity	Energy	0.02163 kWh	market for electricity, medium voltage, renewable energy products | electricity, medium voltage, renewable energy products | Cutoff, U - CH
**Step 2: electrogelation (0.5 mA·cm^{-2} for 20 min)**			
CS/HCl/H_2O_2 solution		100.2000	*From previous step*
For washing	DI water	10.0000	market for water, deionized | water, deionized | Cutoff, U - RoW
Electrogelating electricity	Energy	0.0000642 kWh	market for electricity, medium voltage, renewable energy products | electricity, medium voltage, renewable energy products | Cutoff, U - CH
Part of the solution is reused	Wastewater	25.2763	market for wastewater, average | wastewater, average | Cutoff, U - RoW
From H_2O_2 decomposition	O_2	0.056471 [d]	Emission to air / unspecified
**Step 3: hydrogel immersion (3 M $ZnSO_4$)**			
CS hydrogel at 8.0 wt%		5.2000	*From previous step*
For ionic conductivity	$ZnSO_4·7H_2O$	1.8712 [e]	market for zinc monosulfate | zinc monosulfate | Cutoff, U - RoW
To dissolve	DI water	0.8266 [f]	market for water, deionized | water, deionized | Cutoff, U - RoW
-	Wastewater	2.9252	market for wastewater, average | wastewater, average | Cutoff, U - RoW
**Output material**			

Final material	E-CS-GE	6.1000	-

[a-c]: The remained CS solution without being electrogelated can be recycled and reused for the electrogelation by 5 times (reusing liquid for 4 recycles).

[a]: The amount of CS powder in the first cycle is 1 g, and the amount of new CS in each recycle is 0.416 g (four rounds were tested). Therefore, the amount of new CS considered for each cycle is 0.5328 g.

[b]: HCl can be recycled and reused 5 times (reusing 4 times). 0.0820 g at 37%.

[c]: The recycled solution can continue to dissolve new CS powder. Since the mass of the solution decreases after each treatment, the washing water from step 2 is added to the solution to keep the mass of the solution constant.

[d]: O_2 is generated by the decomposition of H_2O_2. The mass of O_2 = 0.2 g*30%/34*32 = 0.056471 g.

[e-f]: The remaining $ZnSO_4{\cdot}7H_2O$ solution can be recycled and reused for immersion by 5 times (re-using liquid for 4 recycles).

[e]: The amount of $ZnSO_4{\cdot}7H_2O$ in the first cycle is 5.8671 g, and the amount of new $ZnSO_4{\cdot}7H_2O$ in each recycle (4 times) is 2.2815 g, and the amount of $ZnSO_4{\cdot}7H_2O$ for each cycle is 2.9986 g. Equals to 1.8712 g of $ZnSO_4{\cdot}H_2O$.

[f]: DI water can be recycled and reused for the dissolution 5 times (reusing 4 times).

Table S11. Material and energy inventory for "E-CS-GE *recycling*". The outputs from the previous step are highlighted in blue, and wastes are highlighted in light orange.

Comment	Item	Amount (g)	Source/provider
**Step 1: Redissolution**			
	E-CS-GE	5.0000 [a]	*From previous step*
To dissolve CS	HCl solution	0.259	market for hydrochloric acid, without water, in 30% solution state | hydrochloric acid, without water, in 30% solution state | Cutoff, U - RoW
0.2 g at 30% to avoid H_2 bubbles	H_2O_2 solution	0.070	market for hydrogen peroxide, without water, in 50% solution state | hydrogen peroxide, without water, in 50% solution state | Cutoff, U - RoW
To dissolve CS	DI water	130.790	market for water, deionized | water, deionized | Cutoff, U - RoW
Stirring electricity	Energy	0.00742 kWh	market for electricity, medium voltage, renewable energy products | electricity, medium voltage, renewable energy products | Cutoff, U - CH
-	Water steam	97.8133	Emission to air / unspecified
**Output material**			
For further electrogelation. It yields 2.130 g "E-CS-GE" after electrogelation and immersion	CS/HCl/H_2O_2 solution	35.070	-

[a]: A conservative estimate of an 82 wt% loss of the electrolyte material is considered (e.g., CS adhering to zinc anodes, consumption of water and salt, etc.).

The electrolytes are immersed in water to remove the $ZnSO_4$ from the hydrogel electrolyte. The $ZnSO_4$ solution was added to an evaporation unit and concentrated to 3 M $ZnSO_4$ at 100 °C. The CS hydrogel was added to 0.25 wt% HCl solution under stirring for 20 min to obtain the CS solution. Then, 0.072 g of 30 wt% H_2O_2 solution was added to the CS solution. The mixture solution was used to prepare the CS hydrogel.

Table S12. Material and energy inventory for "*CS*". Wastes are highlighted in light orange.

Comment	Item	Amount (kg)	Source/provider
**Step 1: washing and drying at 60 ºC [a]**			
Raw material acquisition	Shrimp shells	35.00	*Burden free* according to *cut-off.* 150 km transport by "market for transport, freight, lorry 3.5-7.5 metric ton, EURO5 | transport, freight, lorry 3.5-7.5 metric ton, EURO5 | Cutoff, U - RoW"
For washing, not determined	DI water	100 L	market for water, deionized | water, deionized | Cutoff, U - RoW
For mechanical agitation	Energy	0.020417 kWh	market for electricity, medium voltage, renewable energy products | electricity, medium voltage, renewable energy products | Cutoff, U - CH
Drying electricity, oven	Energy	0.0008420 kWh	
-	Wastewater	99.00	market for wastewater, unpolluted | wastewater, unpolluted | Cutoff, U – RoW
-	Evaporated water	1.00	Emission to air / unspecified
**Step 2: deproteinization [b]**			
Shell/alkaline solution w/v 1:10, 87.5 L.	NaOH	3.45 *	market for sodium hydroxide, without water, in 50% solution state | sodium hydroxide, without water, in 50% solution state | Cutoff, U – RoW
	DI water	86.27	market for water, deionized | water, deionized | Cutoff, U - RoW
For heating and stirring	Energy	14.281162 kWh	market for electricity, medium voltage, renewable energy products | electricity, medium voltage, renewable energy products | Cutoff, U - CH
Alkaline water	Wastewater	90.00	market for wastewater, average | wastewater, average | Cutoff, U - RoW
**Step 3: demineralization [c]**			
Shell/acid solution w/v 1:15, 84 L	HCl	2.71 *	market for hydrochloric acid, without water, in 30% solution state | hydrochloric acid, without water, in 30% solution state | Cutoff, U - RoW
	DI water	81.00	market for water, deionized | water, deionized | Cutoff, U - RoW
For stirring	Energy	18.1692 kWh	market for electricity, medium voltage, renewable energy products | electricity,

			medium voltage, renewable energy products | Cutoff, U - CH
Acid water	Wastewater	84.00	market for wastewater, average | wastewater, average | Cutoff, U - RoW
**Step 4: decolorization [d]**			
Shell/solution w/v 1:03	Ethanol	3.82	market for ethanol, without water, in 95% solution state, from fermentation | ethanol, without water, in 95% solution state, from fermentation | Cutoff, U - RoW
	Water	0.45	market for water, deionized | water, deionized | Cutoff, U - RoW
For heating and stirring	Energy	0.263592 kWh	market for electricity, medium voltage, renewable energy products | electricity, medium voltage, renewable energy products | Cutoff, U - CH
-	Wastewater	32.2547	market for wastewater, average | wastewater, average | Cutoff, U - RoW
**Step 5: deacetylation**			
Chitin/alkaline solution: w/v 1:10. 11 L	NaOH	7.24	market for sodium hydroxide, without water, in 50% solution state | sodium hydroxide, without water, in 50% solution state | Cutoff, U - RoW
Energy for heating	Energy	3.377752 kWh	market for electricity, medium voltage, renewable energy products | electricity, medium voltage, renewable energy products | Cutoff, U - CH
Drying electricity, oven	Energy	0.0001 kWh	
Alkaline water	Wastewater	10.00	market for wastewater, average | wastewater, average | Cutoff, U - RoW
-	Evaporated water	1.00	Emission to air / unspecified
**Output material**			
Final material	CS	1.00	-

[a]: A 75 wt% loss is considered after this process.

[b]: A 36 wt% loss is considered (protein lost).

[c]: A 32 wt% loss is considered (calcium- and magnesium-based minerals).

[d]: No loss is considered.

*: A density of 1.52 g/cm^3 is estimated for 50% NaOH and 1.145 for 30% HCl when transforming from mass (during the process) to volume (database information).

Table S13. Material and energy inventory for "*E-CMC-GE*". The outputs from the previous step are highlighted in blue, and wastes are highlighted in light orange.

Comment	Item	Amount (g)	Source/provider
**Step 1: CMC solution (100 g solution)**			
Raw materials	CMC	0.6375 [a]	market for carboxymethyl cellulose, powder | carboxymethyl cellulose, powder | Cutoff, U - GLO
To dissolve CMC	DI water	19.8000 [b]	market for water, deionized | water, deionized | Cutoff, U - RoW
Stirring electricity	Energy	0.01442 kWh	market for electricity, medium voltage, renewable energy products | electricity, medium voltage, renewable energy products | Cutoff, U - CH
**Step 2: electrogelation (0.5 mA·cm^{-2} for 20 min)**			
CMC solution		100.0000	*From previous step*
For washing	DI water	10.0000	market for water, deionized | water, deionized | Cutoff, U - RoW
Electrogelating electricity	Energy	0.00004342 kWh	market for electricity, medium voltage, renewable energy products | electricity, medium voltage, renewable energy products | Cutoff, U - CH
Part of the solution is reused	Wastewater	23.5717	market for wastewater, average | wastewater, average | Cutoff, U - RoW
**Step 3: hydrogel crosslinking in $Al_2(SO_4)_3$**			
CMC hydrogel, at 6.6 wt%		6.8658	*From previous step*
For crosslinking	$Al_2(SO_4)_3\cdot 18H_2O$	0.05106 [c]	market for aluminium sulfate, powder | aluminium sulfate, powder | Cutoff, U - RoW
To dissolve	DI water	1.9610 [d *]	market for water, deionized | water, deionized | Cutoff, U - RoW
-	Wastewater	5.6831	market for wastewater, average | wastewater, average | Cutoff, U - RoW
**Step 4: hydrogel immersion (3 M $ZnSO_4$)**			
Crosslinked CMC hydrogel, at 13.9 wt%		3.2485	*From previous step*
For ionic conductivity	$ZnSO_4\cdot 7H_2O$	1.0764 [e]	market for zinc monosulfate | zinc monosulfate | Cutoff, U - RoW

To dissolve	DI water	0.8266 [f]	market for water, deionized | water, deionized | Cutoff, U - RoW
-	Wastewater	3.6496	market for wastewater, average | wastewater, average | Cutoff, U - RoW
**Output material**			
Final material	E-CMC-GE	2.1504	-

[a-b]: The remained CMC solution without being electrogelated can be recycled and reused for the electrogelation by 5 times (re-using liquid for 4 recycles).

[a]: The amount of CMC powder in the first cycle is 1 g, and the amount of new CMC in each recycle is 0.5469 g (four additional rounds). Therefore, the amount of CMC for each cycle is 0.6375 g.

[b]: The recycled CMC solution can continue to dissolve new CS powder. Since the mass of the solution decreases after each treatment, the washing water from step 2 is added to the solution to keep the mass of the solution constant.

[c-d]: The used $Al_2(SO_4)_3$ solution can be recycled and reused for the immersion by 5 times (re-using liquid for 4 recycles).

[c]: The amount of $Al_2(SO_4)_3\cdot18H_2O$ in the first cycle is 0.1948 g, and the amount of new $Al_2(SO_4)_3\cdot18H_2O$ in each recycle (4 times) is 0.0823 g, and the amount of $Al_2(SO_4)_3\cdot18H_2O$ for each cycle is 0.1048 g.

[d]: DI water can be recycled and reused for the dissolvation by 5 times (reusing 4 times).

[e-f]: The remained $ZnSO_4\cdot7H_2O$ solution can be recycled and reused for the immersion by 5 times (re-using liquid for 4 recycles).

[e]: The amount of $ZnSO_4\cdot7H_2O$ in the first cycle is 5.8671 g, and the amount of new $ZnSO_4\cdot7H_2O$ in each recycle (4 times) is 0.6894 g, and the amount of $ZnSO_4\cdot7H_2O$ for each cycle is 1.7250 g. Equals to 1.0764 g of $ZnSO_4\cdot H_2O$.

[f]: DI water can be recycled and reused for the dissolvation by 5 times (reusing 4 times).

*: The database has "$Al_2O_{12}S_3$". Therefore, a conversion from 0.1048 g of $Al_2(SO_4)_3\cdot18H_2O$ needs to be applied.

Table S14. Disaggregated climate change impact for CS and CMC-based electrolytes (a dynamic accounting for biogenic carbon storage is applied, with a lifespan of twenty years).

	Input/output item	E-CS-GE	Re E-CS-GE	E-CMC-GE
Step 1	*CS*	2.243269	-	-
	Carboxymethyl cellulose	-	-	1.28425447
	HCl	0.0163477	0.120264069	-
	H_2O_2	0.0435939	0.07268175	-
	Water	0.0016750	0.033045969	0.004536
	Electricity for dissolution	0.0452844	0.044965776	0.085800
	Biogenic carbon	−0.0160714	−0.0302348	−0.07945
Step 2	*Water*	0.0008496	-	0.0022914
	Electricity for electrogelation	1.2561×10^{-4}	-	2.3802×10^{-4}
	Wastewater	0.0011626	0.00387636	0.0031837
Step 3	*$Al_2(SO_4)_3 \cdot 18H_2O$*	-	-	Negligible
	Water	-	-	0.0004495
	Wastewater	-	-	0.0007676
Step 4	*$ZnSO_4 \cdot 7H_2O$*	0.2423613	0.694256076	0.3955518
	Water	0.0000703	-	0.0001889
	Wastewater	1.348×10^{-4}	-	0.0004929
TOTAL		**2.578803088**	**0.9388552**	**1.6983109**

Table S15. Material and energy inventory for "*PAM/CNF/DMSO electrolyte*".

**Comment**	**Item**	**Amount (g)**	**Source/provider**
**Step 1: dissolution**			
Replaced by polyacrylamide in the database due to lack of these three inputs	Acrylamide	3.000	market for polyacrylamide | polyacrylamide | Cutoff, U - GLO
	Potassium persulfate	0.030	
	N,N′-methylenebis(acrylamide)	0.002	
-	Water	30.000 [a]	market for water, deionized | water, deionized | Cutoff, U - RoW
-	Dimethyl sulfoxide	8.250	market for dimethyl sulfoxide | dimethyl sulfoxide | Cutoff, U - GLO
-	$ZnSO_4$	12.109	market for zinc monosulfate | zinc monosulfate | Cutoff, U - RoW
-	$MnSO_4$	1.133	market for manganese sulfate | manganese sulfate | Cutoff, U - GLO
-	Cellulose nanofibers	0.300	According to: *Journal of Cleaner Production **2020**, 247, 119107*. Utilizes the same LCIA methodology.
Stirring electricity	Energy	0.001577 kWh	market for electricity, medium voltage, renewable energy products | electricity, medium voltage, renewable energy products | Cutoff, U - CH
**Step 2: polymerization**			
Heating electricity	Energy	0.000001703 kWh	market for electricity, medium voltage, renewable energy products | electricity, medium voltage, renewable energy products | Cutoff, U - CH
**Output material**			
-	PAM/CNF/DMSO electrolyte	52.082 [b]	-

[a]: Since the concentration of CNF was not mentioned in the article, we assumed a concentration of 1 wt%. The mass of water is 30 g, and the volume is 30 mL.

[b]: Not stated in the manuscript. Obtained after calculations on mass balance with a 5 wt% loss during the process.

Table S16. Environmental impacts according to *Environmental Footprint v 3.1*.

Impact category	E-CS-GE	Re E-CS-GE	E-CMC-E	PAM/CNF/DMSO	Unit
Acidification	0.017532441	0.01010	0.0120926	0.0582828022	mol H^+-Eq
Climate change	2.578803144	0.9388552	1.698311	5.3326135988	kg CO_2-Eq
Ecotoxicity: freshwater	121.9377554	34.71266	27.0633271	424.7645770749	CTUe
Eutrophication: freshwater	0.00103134	0.00053	0.0007327	0.0046452761	kg P-Eq
Eutrophication: marine	0.004246948	0.00130	0.0019943	0.0056162781	kg N-Eq
Eutrophication: terrestrial	0.041362413	0.01319	0.0199744	0.0488407090	mol N-Eq
Human toxicity: carcinogenic	2.19951E-08	0.00000	0.0000000	0.0000004051	CTUh
Human toxicity: non-carcinogenic	1.72963E-07	0.00000	0.0000002	0.0000014809	CTUh
Material resources: metals/minerals	7.46157E-05	0.00011	0.0000707	0.0000888841	kg Sb-Eq
Water use	7.307704451	4.89927	4.8617391	0.6220908782	m^3 world Eq deprived

Table S17. Main TEA assumptions for the fabrication of "*E-CS-GE*" and "*E-CMC-GE*".

**General assumptions**		
Project start	2024	year
Location	Wuhan, China	
Production volume	360	tons·year^{-1}
Product	*CS electrolyte, CMC electrolyte*	
**Raw material assumptions**		
CS	110000	¥ t^{-1}
Carboxymethyl cellulose	4000	¥ t^{-1}
Water	1.8	¥ t^{-1}
H_2O_2	933	¥ t^{-1}
HCl	85	¥ t^{-1}
$Al_2(SO_4)_3 \cdot 18H_2O$	4000	¥ t^{-1}
$ZnSO_4 \cdot 7H_2O$	4500	¥ t^{-1}
Energy	0.5489	¥ kWh
**Labor and production assumptions**		
Staff	50	workers
Average working days per year	260	days
Shifts per day	1	shift
Hours per turn	8	hour
Direct labor cost	12.5	¥ hour^{-1}
Indirect labor cost	30	%
Fringe coefficient	20	%
Working hours per year per person	2,080	h
Overall equipment effectiveness	90	%
**Property and plant assumptions**		
Capital cost	6.7	%
Maintenance cost	5	%
Insurance cost	1	%
Offsite/Outside Battery Limits (OSBL)	16	%
Engineering cost	20	%
Contingencies	10	%
Years to recover equipment investment	20	year
Years to recover factory investment	30	year
Factory floor required for manufacturing	10000	m^2
Built factory floor space cost	300	¥ m^{-2}
**Equipment assumptions**		
Stirrer	136,000	¥
Automated production equipment for electrogelation	2,800,000	¥
Packing machine	56,000	¥
Other equipment [a]	100,000	¥

[a]: Including automation equipment as required and tools necessary for transporting materials and products.

Table S18. Detailed TEA results for the fabrication of "*E-CS-GE*", "Re *E-CS-GE*", and "*E-CMC-GE*".

	**E-CS-GE**		**Re E-CS-GE**		**E-CMC-GE**	
**Raw material**	**¥ kg^{-1}**	**¥ year^{-1}**	**¥ kg^{-1}**	**¥ year^{-1}**	**¥ kg^{-1}**	**¥ year^{-1}**
Raw material cost per kilo	11.86522	4271479.2	6.515815	2345693.29	5.04075	1814670
Energy cost per kilo	1.951712	702616.32	1.92866	694317.6	3.6916	1328976
**Total raw material costs**	**13.81693**	**4974095.52**	**8.444475**	**3040010.89**	**8.73235**	**3143646**
**Labor**	**¥ kg^{-1}**	**¥ year^{-1}**	**¥ kg^{-1}**	**¥ year^{-1}**	**¥ kg^{-1}**	**¥ year^{-1}**
Direct labor cost	4.012346	1444444.44	4.012346	1444444.44	4.012346	1444444.44
Indirect labor cost	1.203704	433333.333	1.203704	433333.333	1.203704	433333.333
Fringe coefficient	1.04321	375555.556	1.04321	375555.556	1.04321	375555.556
**Total labor costs**	**6.259259**	**2253333.33**	**6.259259**	**2253333.33**	**6.259259**	**2253333.33**
**Property/plant/equipment**	**¥ kg^{-1}**	**¥ year^{-1}**	**¥ kg^{-1}**	**¥ year^{-1}**	**¥ kg^{-1}**	**¥ year^{-1}**
Capital cost	0.028773	10358.2	0.028773	10358.2	0.028773	10358.2
Maintenance	0.021472	7730	0.021472	7730	0.021472	7730
Insurance	0.004294	1546	0.004294	1546	0.004294	1546
Offsite/Outside Battery Limits	0.068711	24736	0.068711	24736	0.068711	24736
Engineering	0.085889	30920	0.085889	30920	0.085889	30920
Contingency	0.042944	15460	0.042944	15460	0.042944	15460
Factory floor space cost	0.277778	100000	0.277778	100000	0.277778	100000
Machinery	0.429444	154600	0.429444	154600	0.429444	154600
**Total property, plant and equipment costs**	**0.959306**	**345350.2**	**0.959306**	**345350.2**	**0.959306**	**345350.2**
**Annual costs**	**7247063.053**		**5312978.425**		**5416613.533**	
**Initial capital**	**7158740**		**7158740**		**7158740**	

**investment**						
**TOTAL PRODUCTIO N COSTS**	**21.035**	**7.57E+06**	**15.663**	**5.64E+06**	**15.951**	**5.74E+06**

References

1. Yang C, Wang MY, Wang W *et al.* Electrodeposition induced covalent cross-linking of chitosan for electrofabrication of hydrogel contact lenses. *Carbohydr Polym* 2022; **292**: 119678.
2. Cheng Y, Cheng G, Xie C *et al.* Biomimetic silk fibroin hydrogels strengthened by silica nanoparticles distributed nanofibers facilitate bone repair. *Adv Healthcare Mater* 2021; **10**: e2001646.
3. Yu L, Huang J, Wang SJ *et al.* Ionic liquid "water pocket" for stable and environment-adaptable aqueous zinc metal batteries. *Adv Mater* 2023; **35**: 2210789.
4. Jian QP, Wang TS, Sun J *et al.* In-situ construction of fluorinated solid-electrolyte interphase for highly reversible zinc anodes. *Energy Storage Mater* 2022; **53**: 559–68.
5. Ma GQ, Miao LC, Yuan WT *et al.* Non-flammable, dilute, and hydrous organic electrolytes for reversible Zn batteries. *Chem Sci* 2022; **13**: 11320–9.
6. Lu T, Chen F. Multiwfn: A multifunctional wavefunction analyzer. *J Comput Chem* 2011; **33**: 580–92.
7. Ma Y, Zhang Q, Liu L *et al.* N,N-dimethylformamide tailors solvent effect to boost Zn anode reversibility in aqueous electrolyte. *Natl Sci Rev* 2022; **9**: nwac051.
8. Li PF, Roberts BP, Chakravorty DK *et al.* Rational design of particle mesh ewald compatible lennard-jones parameters for +2 metal cations in explicit solvent. *J Chem Theory Comput* 2013; **9**: 2733–48.
9. Miao LC, Wang RH, Di SL *et al.* Aqueous electrolytes with hydrophobic organic cosolvents for stabilizing zinc metal anodes. *ACS Nano* 2022; **16**: 9667–78.
10. Laurent A, Weidema BP, Bare J *et al.* Methodological review and detailed guidance for the life cycle interpretation phase. *J Ind Ecol* 2020; **24**: 986–1003.
11. Li ZH, Chen CJ, Xie H *et al.* Sustainable high-strength macrofibres extracted from natural bamboo. *Nat Sustain* 2022; **5**: 235–44.
12. Xiao SL, Chen CJ, Xia QQ *et al.* Lightweight, strong, moldable wood via cell wall engineering as a sustainable structural material. *Science* 2021; **374**: 465-71.
13. Xia Q, Chen C, Yao Y *et al.* A strong, biodegradable and recyclable lignocellulosic bioplastic. *Nat Sustain* 2021; **4**: 627-35.

14. Riofrio A, Alcivar T, Baykara H. Environmental and economic viability of chitosan production in guayas-ecuador: a robust investment and life cycle analysis. *ACS Omega* 2021; **6**: 23038–51.

15. Sudheshwar A, Vogel K, Nyström G *et al.* Unraveling the climate neutrality of wood derivatives and biopolymers. *RSC Sustain* 2024; **2**: 1487–97.

16. de Assis CA, Greca LG, Ago M *et al.* Techno-economic assessment, scalability, and applications of aerosol lignin micro- and nanoparticles. *ACS Sustainable Chem Eng* 2018; **6**: 11853–68.

17. Chen L, Bi T, Lizundia E *et al.* Biomass waste-assisted micro(nano)plastics capture, utilization, and storage for sustainable water remediation. *The Innovation* 2024; **5**: 100655.

18. Zhang ZC, Wang XW, Ke JQ *et al.* Approaching 100% Comprehensive Utilization Rate of Ultra-Stable Zn Metal Anodes by Constructing Chitosan-Based Homologous Gel/Solid Synergistic Interface. *Adv Funct Mater* 2024; **34**.

19. Wang YF, Zhang Z, Wang M *et al.* Direct electrodeposition of carboxymethyl cellulose based on coordination deposition method. *Cellulose* 2018; **25**: 105–15.

20. Cheng Y, Luo XL, Betz J *et al.* Mechanism of anodic electrodeposition of calcium alginate. *Soft Matter* 2011; **7**: 5677–84.

21. Bressner JE, Marelli B, Qin GK *et al.* Rapid fabrication of silk films with controlled architectures electrogelation. *J Mater Chem B* 2014; **2**: 4983–7.
